# Supplementary material for: Thirteen dubious ways to detect conserved structural RNAs
Source: IUBMB Life. Author manuscript; Available in PMC 2024 Jul 10. (PMC11234323; doi:10.1002/iub.2694)
Supplement: supplementary_material [file NIHMS1907110-supplement-supplementary_material.gz › supplemental_material/rscape_v2.0.0.k.tar.gz!/rscape_v2.0.0.k/lib/R2R/R2R-current/NotByZasha/nlopt-2.6.2/src/algs/luksan/v999-07.pdf]

Institute of Computer Science
Academy of Sciences of the Czech Republic

New subroutines for large-scale optimization

Ladislav Lukšan, Ctirad Matonoha, Jan Vlček

Technical report No. V999

June 20, 2007

Pod Vodárenskou věží 2, 182 07 Prague 8 phone: +420 2 688 42 44, fax: +420 2 858 57 89,
e-mail:e-mail:ics@cs.cas.cz

Institute of Computer Science
Academy of Sciences of the Czech Republic

New subroutines for large-scale optimization

Ladislav Lukšan, Ctirad Matonoha, Jan Vlček [1]

Technical report No. V999

June 20, 2007

Abstract:

We present fourteen basic FORTRAN subroutines for large-scale optimization with simple bounds and large-scale systems of nonlinear equations. Subroutines `PLIS` and `PLIP`, intended for dense general optimization problems, are based on limited-memory variable metric methods. Subroutine `PNET`, also intended for dense general optimization problems, is based on an inexact truncated Newton method. Subroutines `PNED` and `PNEC`, intended for sparse general optimization problems, are based on modifications of the discrete Newton method. Subroutines `PSED` and `PSEC`, intended for partially separable optimization problems, are based on partitioned variable metric updates. Subroutine `PSEN`, intended for nonsmooth partially separable optimization problems, is based on partitioned variable metric updates and on an aggregation of subgradients. Subroutines `PGAD` and `PGAC`, intended for sparse nonlinear least squares problems, are based on modifications and corrections of the Gauss-Newton method. Subroutine `PMAX`, intended for minimization of a maximum value (minimax), is based on the primal line-search interior-point method. Subroutine `PSUM`, intended for minimization of a sum of absolute values, is based on the primal trust-region interior-point method. Subroutines `PEQN` and `PEQL`, intended for sparse systems of nonlinear equations, are based on the discrete Newton method and the inverse column-update quasi-Newton method, respectively. Besides the description of methods and codes, we propose computational experiments which demonstrate the efficiency of the proposed algorithms.

Keywords:
Large-scale optimization, Large-scale nonsmooth optimization, large-scale nonlinear least squares, large-scale nonlinear minimax, large-scale systems of nonlinear equations, sparse problems, partially separable problems, limited-memory methods, discrete Newton methods, quasi-Newton methods, primal interior-point methods.

[1] This work was supported by the Grant Agency of the Czech Academy of Sciences, project No. IAA1030405, the Grant Agency of the Czech Republic, project No. 201/06/P397, and the institutional research plan No. AV0Z10300504

1 Introduction

We propose fourteen basic subroutines which implement selected large-scale optimization algorithms. The double-precision FORTRAN 77 subroutines `PLIS`, `PLIP`, `PNET` are designed to find a close approximation to a local minimum of a general twice continuously differentiable function $F : R^n \to R$ with unknown or dense Hessian matrix. Subroutines `PLIS`, `PLIP` are based on limited-memory variable metric updates: `PLIS` uses Strang recurrences [13], [26] and `PLIP` uses shifted limited-memory variable metric updates [38]. Subroutine `PNET` is based on an inexact truncated Newton method [8].

The double-precision FORTRAN 77 subroutines `PNED`, `PNEC` are designed to find a close approximation to a local minimum of a general twice continuously differentiable function $F : R^n \to R$ with the sparse Hessian matrix. These subroutines are based on inexact discrete Newton methods [5], [7]: `PNED` uses matrix direct methods (Dennis–Mei [9] or Moré–Sorensen [25]) and `PNEC` uses matrix iterative methods (Steihaug–Toint [31], [32] or shifted Steihaug–Toint [16]) for computing a trust region step.

The double-precision FORTRAN 77 subroutines `PSED`, `PSEC` are designed to find a close approximation to a local minimum of a smooth partially separable objective function

$$F(x) = \sum_{i=1}^{n_a} f_i(x).$$

Here $x \in R^n$ is a vector of n variables and $f_i : R^n \to R$, $1 \le i \le n_a$, are twice continuously differentiable functions. These subroutines are based on partitioned variable metric updates [12], [19]: `PSED` uses a matrix direct method (Gill-Murray [10]) and `PSEC` uses a matrix iterative method (safeguarded conjugate gradient method) for computing a line-search step.

The double-precision FORTRAN 77 subroutine `PSEN` is designed to find a close approximation to a local minimum of a nonsmooth partially separable objective function

$$F(x) = \sum_{i=1}^{n_a} f_i(x).$$

Here $x \in R^n$ is a vector of n variables and $f_i : R^n \to R$, $1 \le i \le n_a$, are locally Lipschitz nondifferentiable functions. This subroutine is based on a bundle variable metric method described in [23].

The double-precision FORTRAN 77 subroutines `PGAD`, `PGAC` are designed to find a close approximation to a local minimum of the least-square function

$$F(x) = \frac{1}{2} \sum_{i=1}^{n_a} f_i^2(x).$$

Here $x \in R^n$ is a vector of n variables and $f_i : R^n \to R$, $1 \le i \le n_a$, are twice continuously differentiable functions. These subroutines are based on hybrid methods that combine the Gauss-Newton method with the Newton or the variable metric corrections [15], [19]: `PGAD` uses matrix direct methods (Dennis–Mei [9] or Moré–Sorensen [25]) and `PGAC` uses matrix iterative methods (Steihaug–Toint [31], [32] or shifted Steihaug–Toint [16]) for computing a trust region step.

The double-precision FORTRAN 77 subroutine PMAX is designed to find a close approximation to a local minimum of maximum functions

$$F(x) = \max_{1 \le i \le n_a} f_i(x)$$

or

$$F(x) = \max_{1 \le i \le n_a} |f_i(x)|.$$

Here $x \in R^n$ is a vector of n variables and $f_i : R^n \to R$, $1 \le i \le n_a$, are twice continuously differentiable functions. This subroutine is based on primal interior point methods described in [17].

The double-precision FORTRAN 77 subroutine PSUM is designed to find a close approximation to a local minimum of a sum of absolute values

$$F(x) = \sum_{i=1}^{n_a} |f_i(x)|.$$

Here $x \in R^n$ is a vector of n variables and $f_i : R^n \to R$, $1 \le i \le n_a$, are twice continuously differentiable functions. This subroutine is based on primal interior point methods described in [18].

The double-precision FORTRAN 77 subroutines PEQN, PEQL are designed to find a solution of a system of nonlinear equations

$$f_i(x) = 0, \qquad 1 \le i \le n.$$

Here $x \in R^n$ is a vector of n variables and $f_i : R^n \to R$, $1 \le i \le n$, are continuously differentiable functions. Subroutine PEQN is based on the inexact discrete Newton method [2], [6], [22] and subroutine PEQL is based on the inverse column-update quasi-Newton method [22], [24].

Subroutines PLIS, PLIP, PNET, PNED, PNEC, PSED, PSEC, PGAD, PGAC allow us to work with box constraints in the form

$$\begin{aligned}
x_i - \text{unbounded} \quad &, \quad I_i^x = 0, \\
x_i^l \le x_i \quad &, \quad I_i^x = 1, \\
x_i \le x_i^u \quad &, \quad I_i^x = 2, \\
x_i^l \le x_i \le x_i^u \quad &, \quad I_i^x = 3, \\
x_i - \text{fixed} \quad &, \quad I_i^x = 5,
\end{aligned}$$

where $1 \le i \le n$ (I^x corresponds to array IX in Appendix A).

To simplify the user's work, additional easy-to-use subroutines are added. These subroutines, written in FORTRAN 90 (they use a dynamic allocation of working arrays), call general subroutines PLIS, PLIP, PNET, PNED, PNEC, PSED, PSEC, PSEN, PGAD, PGAC, PMAX, PSUM, PEQN, PEQL. These subroutines can be easily changed to FORTRAN 77 codes by deleting statements ALLOCATABLE, ALLOCATE, DEALLOCATE, adding working arrays IA, RA into formal parameters and declaring their dimensions in the main program. Table 1 summarizes properties of easy-to-use subroutines.

Each subroutine contains a description of formal parameters and extensive comments. Moreover, text files PLIS.TXT, PLIP.TXT, PNET.TXT, PNED.TXT, PNEC.TXT, PSED.TXT,

Subroutine	Problem	Constraints	Strategy	Matrices	Solver
PLISU	general	no	line-search	no	no
PLISS	general	yes	line-search	no	no
PLIPU	general	no	line-search	no	no
PLIPS	general	yes	line-search	no	no
PNETU	general	no	line-search	no	iterative
PNETS	general	yes	line-search	no	iterative
PNEDU	general	no	trust-region	yes	direct
PNEDS	general	yes	trust-region	yes	direct
PNECU	general	no	trust-region	yes	iterative
PNECS	general	yes	trust-region	yes	iterative
PSEDU	partially separable	no	line-search	yes	direct
PSEDS	partially separable	yes	line-search	yes	direct
PSECU	partially separable	no	line-search	yes	iterative
PSECS	partially separable	yes	line-search	yes	iterative
PSENU	nonsmooth	no	line-search	yes	direct
PGADU	sum of squares	no	trust-region	yes	direct
PGADS	sum of squares	yes	trust-region	yes	direct
PGACU	sum of squares	no	trust-region	yes	iterative
PGACS	sum of squares	yes	trust-region	yes	iterative
PMAXU	minimax and l_∞	no	line-search	yes	direct
PSUMU	l_1	no	trust-region	yes	direct
PEQNU	nonlinear equations	no	line-search	yes	iterative
PEQLU	nonlinear equations	no	line-search	yes	iterative

Table 1: Easy-to-use subroutines

PSEC.TXT, PSEN.TXT, PGAD.TXT, PGAC.TXT, PMAX.TXT, PSUM.TXT, PEQN.TXT, PEQL.TXT containing a detailed description of all important subroutines are added. Finally, test programs TLISU, TLISS, TLIPU, TLIPS, TNETU, TNETS, TNEDU, TNEDS, TNECU, TNECS, TSEDU, TSEDS, TSECU, TSECS, TSENU, TGADU, TGADS, TGACU, TGACS, TMAXU, TSUMU, TEQNU, TEQLU that use extensive collections of test problems are included. These test programs serve as examples for using the subroutines, verify their correctness and demonstrate their efficiency.

Subroutines presented in this report were generated using the UFO system [20]. We selected the most efficient methods from this system to prepare individual problem-oriented subroutines. This point of view gives a reason for our selection of optimization methods and choice of line-search or trust-region strategies (the UFO system contains many additional methods and strategies which were also tested and compared). This also explains the fact that PNET uses line-search while PNED, PNEC use trust-region strategies and the fact that some methods and strategies are implemented in slightly different ways than those described in the original papers (we have carefully tuned them to obtain the best results). Subroutines PLIS, PNET, PNED, PNEC, PSED, PSEC, PGAD, PGAC, PEQN, PEQL implement classic optimization methods and have equivalents, e.g., L-BFGS [40], TNPACK [30], VE08 [33], VE10 [34], NITSOL [28], NLEQ1S [27]. Numerical comparisons with these equivalents are given in Section 9 (we also used HOMPACK90 [39] for a comparison, but this code

was unable to solve a large number of our test problems). Subroutines PLIP, PSEN, PMAX, PSUM are based on new original methods proposed in [38], [23], [17], [18].

All subroutines described in this report are free for academic use and can be downloaded from www.cs.cas.cz/~luksan/subroutines.html.

2 Matrix-free methods for general problems

Consider a general continuously differentiable function $F : R^n \to R$, where n is large, and assume that the structure of the Hessian matrix of F is unknown. In this case, subroutines PLIS, PLIP, and PNET based on matrix-free methods can be used for seeking a local minimum of F. These methods are realized in the line-search framework so that they generate a sequence of points $x_k \in R^n$, $k \in N$, by the simple process

$$x_{k+1} = x_k + \alpha_k d_k, \tag{1}$$

where $d_k \in R^n$ is a direction vector and $0 < \alpha_k \le \overline{\alpha}_k$ is a scalar step-size. The direction vector is determined in such a way that

$$-d_k^T g(x_k) \ge \underline{\varepsilon} \|d_k\| \|g(x_k)\| \tag{2}$$

(the uniform descent condition) holds, where $0 < \underline{\varepsilon} \le 1$ (we use the value $\underline{\varepsilon} = 10^{-4}$ in our subroutines). The step-size is chosen in such a way that

$$F(x_{k+1}) - F(x_k) \le \varepsilon_1 \, \alpha_k \, d_k^T g(x_k), \quad d_k^T g(x_{k+1}) \ge \varepsilon_2 \, d_k^T g(x_k) \tag{3}$$

(the weak Wolfe conditions) hold, where $0 < \varepsilon_1 < 1/2$ is a tolerance for the function value decrease and $\varepsilon_1 < \varepsilon_2 < 1$ is a tolerance for the directional derivative increase (we use the values $\varepsilon_1 = 0.0001$ and $\varepsilon_2 = 0.9$ in our subroutines). The maximum step-size $\overline{\alpha}_k$ is given by the formula $\overline{\alpha}_k = \overline{\Delta}/\|d_k\|$, where $\overline{\Delta}$ is an upper bound for the norm $\|x_{k+1} - x_k\|$ (parameter XMAX in our subroutines). The step-size α_k is chosen iteratively either by bisection (MES $= 1$), or by quadratic interpolation with two function values (MES $= 2$), or by quadratic interpolation with two directional derivatives (MES $= 3$), or by cubic interpolation (MES $= 4$). We start with the initial estimate $\alpha_k = 1$ if IEST $= 0$ or the initial estimate is derived by using the lower bound for F (parameter FMIN in our subroutines) if IEST $= 1$.

The direction vector d_k is usually computed by the formula $d_k = -H_k g_k$ or by solving the linear system $B_k d_k = -g_k$, where $g_k = g(x_k)$, B_k is an approximation of the Hessian matrix $G(x_k)$ and H_k is an approximation of its inverse. Limited-memory variable metric methods use the matrix H_k implicitly (it is not stored). Similarly, the truncated Newton method uses the matrix B_k, which is not stored as well. If the direction vector computed by using the above way does not satisfy the uniform descent condition (2), a restart is performed, which implies that the direction vector $d_k = -g_k$, satisfying (2), is used.

2.1 Limited-memory BFGS method

Subroutine PLIS is an implementation of the limited-memory BFGS method proposed in [13], [26]. This method works with matrices $H_k = H_k^k$, where $H_{k-m}^k = \gamma_k I$, $\gamma_k > 0$ and

$$H_{j+1}^k = V_j^T H_j^k V_j + \frac{1}{b_j} s_j s_j^T, \qquad V_j = I - \frac{1}{b_j} y_j s_j^T$$

for $k-m \le j \le k-1$. Here $s_j = x_{j+1} - x_j$, $y_j = g_{j+1} - g_j$, $a_j = y_j^T H_j^k y_j$, $b_j = y_j^T s_j$. Thus

$$H_{j+1}^k = \frac{b_{k-1}}{a_{k-1}} \left(\prod_{i=k-m}^{j} V_i \right)^T \left(\prod_{i=k-m}^{j} V_i \right) + \sum_{l=k-m}^{j} \frac{1}{b_l} \left(\prod_{i=l+1}^{j} V_i \right)^T s_l s_l^T \left(\prod_{i=l+1}^{j} V_i \right)$$

(we use $\gamma_k = b_{k-1}/a_{k-1}$ in our implementation). The matrix $H_k = H_k^k$ need not be constructed explicitly since we need only a vector $d_k = -H_k^k g_k$, which can be computed by using two recurrences (the Strang formula). First, vectors

$$u_j = -\left(\prod_{i=j}^{k-1} V_i \right) g_k,$$

$k-1 \ge j \ge k-m$, are computed by using the backward recurrence

$$\begin{aligned} \sigma_j &= s_j^T u_{j+1}/b_j, \\ u_j &= u_{j+1} - \sigma_j y_j, \end{aligned}$$

where $u_k = -g_k$. Then vectors

$$v_{j+1} = \frac{b_{k-1}}{a_{k-1}} \left(\prod_{i=k-m}^{j} V_i \right)^T u_{k-m} + \sum_{l=k-m}^{j} \frac{1}{b_l} \left(\prod_{i=l+1}^{j} V_i \right)^T s_l s_l^T u_{l+1},$$

$k-m \le j \le k-1$, are computed by using the forward recurrence

$$v_{j+1} = v_j + (\sigma_j - y_j^T v_j/b_j) s_j,$$

where $v_{k-m} = (b_{k-1}/a_{k-1}) u_{k-m}$. Finally, we set $d_k = v_k$. Note that $2m$ vectors s_j, y_j, $k-m \le j \le k-1$ are used and stored. The number of consecutive variable metric updates is defined as $m = \min(\mathtt{MF}, k - \underline{k})$, where `MF` is a parameter of the subroutine `PLIS` and $\underline{k}$ is an index of the iteration corresponding to the last restart.

2.2 Shifted limited-memory variable metric methods

Subroutine `PLIP` is an implementation of shifted limited-memory variable metric methods proposed in [38]. These methods work with matrices $H_k = \zeta_k I + U_k U_k^T$, where $n \times m$ matrix U_k is updated by formula $U_{k+1} = V_k U_k$ with a low rank matrix V_k chosen in such a way that the (modified) quasi-Newton condition $U_{k+1} U_{k+1}^T y_k = \rho_k \tilde{s}_k$ with $\tilde{s}_k = s_k - \zeta_{k+1} y_k$ is satisfied (we use the same notation, namely s_k, y_k, a_k, b_k as in Section 2.1). This condition can be replaced by equations

$$U_{k+1}^T y_k = z_k, \qquad U_{k+1} z_k = \rho_k \tilde{s}_k, \qquad \|z_k\|^2 = \rho_k y_k^T \tilde{s}_k.$$

where z_k is an optional vector parameter. Note that the last equality, which is a consequence of the first two equalities, is the only restriction laid on the vector z_k. To simplify the notation, we define vectors $u_k = U_k^T y_k$ and $v_k = U_k^T H_k^{-1} s_k = -\alpha_k U_k^T g_k$.

The choice of a shift parameter ζ_{k+1} is a crucial part of shifted limited-memory variable metric methods. The value

$$\zeta_{k+1} = \mu_k \frac{b_k}{\|y_k\|^2}, \qquad \mu_k = \frac{\sqrt{1 - \|u_k\|^2/a_k}}{1 + \sqrt{1 - b_k^2/(\|s_k\|^2 \|y_k\|^2)}}$$

is used in subroutine PLIP. The most efficient shifted limited-memory variable metric methods can be derived by a variational principle. Let T_k be a symmetric positive definite matrix. It can be shown (see [38]) that the Frobenius norm $\|T_k^{-1/2}(U_{k+1}-U_k)\|_F^2$ is minimal on the set of all matrices satisfying the quasi-Newton condition if and only if

$$U_{k+1} = U_k - \frac{T_k y_k}{y_k^T T_k y_k} y_k^T U_k + \left(\rho_k \tilde{s}_k - U_k z_k + \frac{y_k^T U_k z_k}{y_k^T T_k y_k} T_k y_k \right) \frac{z_k^T}{\|z_k\|^2}.$$

Here $T_k y_k$ and z_k are vector parameters defining a class of shifted limited-memory variable metric methods. Using suitable values of these vectors, we obtain particular methods of this class.

Assuming that $T_k y_k$ and $\rho_k \tilde{s}_k - U_k z_k$ are linearly dependent and setting

$$z_k = \vartheta_k v_k, \qquad \vartheta_k = \pm\sqrt{\rho_k y_k^T \tilde{s}_k / \|v_k\|^2}$$

we obtain rank 1 variationally derived method (VAR1), where

$$U_{k+1} = U_k - \frac{\rho_k \tilde{s}_k - \vartheta_k U_k v_k}{\rho_k y_k^T \tilde{s}_k - \vartheta_k u_k^T v_k} \left(u_k - \vartheta_k v_k \right)^T ,$$

which gives the best results for the choice $\mathrm{sgn}(\vartheta_k u_k^T v_k) = -1$. Method VAR1 is chosen if MET $= 1$ in the subroutine PLIP. Using z_k given above and setting $T_k y_k = \tilde{s}_k$, which corresponds to the BFGS method in the full-memory case, we obtain rank 2 variationally derived method (VAR2), where

$$U_{k+1} = U_k - \frac{\tilde{s}_k}{y_k^T \tilde{s}_k} u_k^T + \left(\rho_k \frac{\tilde{s}_k}{\vartheta_k} - U_k v_k + \frac{u_k^T v_k}{y_k^T \tilde{s}_k} \tilde{s}_k \right) \frac{v_k^T}{\|v_k\|^2}.$$

Method VAR2 is chosen if MET $= 2$ in the subroutine PLIP. The efficiency of both these methods depends on the value of the correction parameter ρ_k. This value is determined by the formula $\rho_k = \sqrt{\nu_k \varepsilon_k}$. Here $\nu_k = \mu_k/(1-\mu_k)$, μ_k is a relative shift parameter defined above and

$$\varepsilon_k = \sqrt{1 - \|u_k\|^2 / a_k}$$

is a damping factor of μ_k. The number of columns of the matrix U_k is defined as $m = \min(\mathrm{MF}, k - \underline{k})$, where MF is a parameter of the subroutine PLIP and $\underline{k}$ is an index of the iteration corresponding to the last restart.

2.3 Inexact truncated Newton method

Subroutine PNET is based on a line-search realization of the Newton method, which uses conjugate gradient (CG) iterations for solving a system of linear equations $G(x)d = -g(x)$ ($g(x)$ and $G(x)$ are the gradient and the Hessian matrix of function $F : R^n \to R$ at the point x, respectively). Since the matrix $G(x)$ can be indefinite, a modification of the (possibly preconditioned) CG method is used, which terminates if a negative curvature is detected. More precisely, we set $d_1 = 0$, $g_1 = g(x)$, $h_1 = C^{-1} g_1$, $p_1 = -h_1$, $\sigma_1 = g_1^T h_1$ and for $i = 1, 2, 3, \ldots$ we proceed in the following way. If $\|g_i\| \le \overline{\omega}(x) \|g(x)\|$, then set $d = d_i$ and terminate the computation, otherwise set

$$q_i = G(x) p_i, \qquad \tau_i = p_i^T q_i.$$

If $\tau_i < \underline{\tau}$ then set $d = -g(x)$ (if $i = 1$) or $d = d_i$ (if $i > 1$) and terminate the computation, otherwise set $\alpha_i = \sigma_i/\tau_i$ and compute

$$\begin{aligned} d_{i+1} &= d_i + \alpha_i p_i, \\ g_{i+1} &= g_i + \alpha_i q_i, \\ h_{i+1} &= C^{-1} g_{i+1}, \qquad \sigma_{i+1} = g_{i+1}^T h_{i+1} \\ p_{i+1} &= -h_{i+1} + (\sigma_{i+1}/\sigma_i) p_i. \end{aligned}$$

The principal feature of the inexact truncated Newton method is the fact that the Hessian matrix $G(x)$ is not used explicitly, but the vector $G(x)p_i$ is computed by numerical differentiation using the formula

$$G(x)p_i \approx \frac{g(x + \delta_i p_i) - g(x)}{\delta_i}, \tag{4}$$

where $\delta_i = \sqrt{\varepsilon_M}/\|p_i\|$ (ε_M is the machine precision). Thus one extra gradient evaluation is needed in every CG iteration.

The CG method is terminated if $\tau_i < \underline{\tau}$ (a negative curvature is detected) or if $\|g_i\| \le \overline{\omega}(x)\|g(x)\|$ (a sufficient precision is achieved). We use the value $\underline{\tau} = 10^{-60}$ in subroutine `PNET`. The value $\overline{\omega}(x)$ is chosen according to the inexact Newton approach [7]. In the k-th Newton iteration we use the value

$$\overline{\omega}(x_k) = \min\left(\sqrt{\|g(x_k)\|}, 1/k, \overline{\omega}\right), \tag{5}$$

where $\overline{\omega} = 0.8$ in subroutine `PNET`

The matrix C serving as a preconditioner (symmetric and positive definite) cannot be derived from the Hessian matrix, which is not known explicitly. If `MOS2` $=$ `1` (`MOS2` is a parameter of the subroutine `PNET`), the unpreconditioned CG method ($C = I$) is used. If `MOS2` $=$ `2`, we use the preconditioner obtained by the limited memory BFGS method. In this case $C^{-1} = H_k = H_k^k$, where H_k^k is a matrix defined in Subsection 2.1. This matrix need not be constructed explicitly, since we need only the vector $h_i = C^{-1} g_i = H_k^k g_i$ (i is an inner index of the CG method), which can be computed by using two recurrences (the Strang formula). First, vectors u_j, $k-1 \ge j \ge k-m$, are computed by using the backward recurrence

$$\begin{aligned} \sigma_j &= s_j^T u_{j+1}/b_j, \\ u_j &= u_{j+1} - \sigma_j y_j, \end{aligned}$$

where $u_k = g_i$. Then vectors v_{j+1}, $k-m \le j \le k-1$, are computed by using the forward recurrence

$$v_{j+1} = v_j + (\sigma_j - y_j^T v_j/b_j) s_j,$$

where $v_{k-m} = (b_{k-1}/a_{k-1}) u_{k-m}$. Finally, we set $h_i = v_k$. Note that $2m$ additional vectors $s_j = x_{j+1} - x_j$, $y_j = g_{j+1} - g_j$, $k-m \le j \le k-1$, has to be stored if `MOS2` $=$ `2`.

2.4 Active set strategy for box constraints

If box constraints are considered, then a simple active set strategy is used. To simplify the notation, we omit iteration index k in the following description. Every iteration is started by the detection of new candidates for active constraints. Thus we set

$$\begin{array}{rlll} x_i = x_i^l, & I_i^x = -1 & \text{if} & I_i^x = 1, \quad x_i \le x_i^l + \varepsilon_c \max(|x_i^l|, 1), \\ x_i = x_i^u, & I_i^x = -2 & \text{if} & I_i^x = 2, \quad x_i \ge x_i^u - \varepsilon_c \max(|x_i^u|, 1), \\ x_i = x_i^l, & I_i^x = -3 & \text{if} & I_i^x = 3, \quad x_i \le x_i^l + \varepsilon_c \max(|x_i^l|, 1), \\ x_i = x_i^u, & I_i^x = -4 & \text{if} & I_i^x = 3, \quad x_i \ge x_i^u - \varepsilon_c \max(|x_i^u|, 1), \\ & I_i^x = -5 & \text{if} & I_i^x = 5 \end{array}$$

for $1 \le i \le n$, where ε_c is a required precision (we use value $\varepsilon_c = 10^{-8}$ in our subroutines). After computing gradient $g = g(x)$, we determine projected gradient g^p and chopped gradient g^c in such a way that

$$\begin{array}{rlll} g_i^p = 0, & g_i^c = \max(0, g_i) & \text{for} & I_i^x = -1 \quad \text{or} \quad I_i^x = -3, \\ g_i^p = 0, & g_i^c = \min(0, g_i) & \text{for} & I_i^x = -2 \quad \text{or} \quad I_i^x = -4, \\ g_i^p = 0, & g_i^c = 0 & \text{for} & I_i^x = -5, \\ g_i^p = g_i, & g_i^c = 0 & \text{for} & I_i^x \ge 0. \end{array}$$

If $\|g^c\|_\infty > \|g^p\|_\infty$ and the previous step was successful, we delete redundant active constraints by setting

$$\begin{array}{llll} I_i^x = 1 & \text{if} & I_i^x = -1 & \text{and} \quad g_i > 0, \\ I_i^x = 2 & \text{if} & I_i^x = -2 & \text{and} \quad g_i < 0, \\ I_i^x = 3 & \text{if} & I_i^x = -3 & \text{and} \quad g_i > 0, \\ I_i^x = 3 & \text{if} & I_i^x = -4 & \text{and} \quad g_i < 0. \end{array}$$

In this way, we have obtained a current set of active constraints for the direction determination, step-size selection and variable metric update. This active set defines a reduced problem with variables x_i, $I_i^x < 0$, fixed. If we introduce matrix Z containing columns e_i, $I_i^x \ge 0$ (e_i is the i-th column of the unit matrix), we can define reduced gradient $g^r = Z^T g$ and reduced matrices H^r or B^r to obtain reduced direction vectors $d^r = -H^r g^r$ or $B^r d^r = -g^r$. Finally, we use the direction vector $d = Z d^r$. In this way, we can adapt an arbitrary line-search or trust-region method to solve the reduced problem. Since the set of active constraints can change, we have to use a suitable restart strategy (conditions for setting $B^r = I$, $H_r = I$ or $d^r = -g^r$). To guarantee descent, the restart is always performed when more then one redundant active constraint is deleted. The preconditioned truncated Newton method is restarted after every change of the set of active constraints.

From a practical point of view, it is not advantageous to construct reduced quantities. A better way is to construct projected gradients $g^p = ZZ^T g$ and projected matrices $H^p = ZZ^T H Z Z^T + YY^T$ or $B^p = ZZ^T B Z Z^T + YY^T$, where Y contains columns e_i, $I_i^x < 0$, to obtain direction vectors $d = -H^p g^p$ or $B^p d = -g^p$. Matrices H^p or B^p are block diagonal (with blocks H^r, I or B^r, I) and they can be updated by using projected vectors $s^p = ZZ^T s = ZZ^T(x^+ - x)$ and $y^p = ZZ^T(g^+ - g)$. Thus it suffices to use projected quantities instead of standard ones. Diagonal blocks of matrices H^p or B^p can

be easily derived from their sparsity pattern by considering only the elements H_{ij}^p or B_{ij}^p for which $I_i^x \geq 0$, $I_j^x \geq 0$ hold simultaneously. These blocks are then used in matrix multiplications and matrix decompositions.

Before step-size selection, we have to determine the maximum step-size $\overline{\alpha}$ to assure the feasibility. This is computed by the formula $\overline{\alpha} = \min(\overline{\alpha}_1, \overline{\alpha}_2, \overline{\alpha}_3, \overline{\alpha}_4, \overline{\Delta}/\|d\|)$, where

$$\overline{\alpha}_1 = \min_{I_i^x=1, d_i<0} \frac{x_i^l - x_i}{d_i}, \qquad \overline{\alpha}_2 = \min_{I_i^x=2, d_i>0} \frac{x_i^u - x_i}{d_i},$$

$$\overline{\alpha}_3 = \min_{I_i^x=3, d_i<0} \frac{x_i^l - x_i}{d_i}, \qquad \overline{\alpha}_4 = \min_{I_i^x=3, d_i>0} \frac{x_i^u - x_i}{d_i}$$

(if a corresponding set is empty we use the value ∞).

3 Inexact discrete Newton methods for sparse problems

Consider a general twice continuously differentiable function $F : R^n \to R$, where n is large, and assume that the Hessian matrix $G(x) = [G_{ij}(x)] = [\partial^2 F(x)/(\partial x_i \partial x_j)]$ is sparse. In this case, discrete versions of the Newton method can be efficiently used for seeking a local minimum of F. These methods are based on the fact that sufficiently sparse Hessian matrices can be estimated by using a small number of gradient differences [5]. We use the algorithm proposed in [36] in subroutines `PNED`, `PNEC`. The sparsity pattern of the Hessian matrix (only the upper part) is stored in the coordinate form (if `ISPAS` = `1`) or in the standard compressed row format (if `ISPAS` = `2`) using arrays `IH` and `JH`. For example, if the Hessian matrix has the pattern

$$G = \begin{bmatrix} * & * & * & 0 & * \\ * & * & 0 & * & 0 \\ * & 0 & * & 0 & * \\ 0 & * & 0 & * & 0 \\ * & 0 & * & 0 & * \end{bmatrix}$$

(asterisks denote nonzero elements), then arrays `IH` and `JH` contain elements

$$\texttt{IH} = \begin{bmatrix} 1 & 1 & 1 & 1 & 2 & 2 & 3 & 3 & 4 & 5 \end{bmatrix}, \quad \texttt{JH} = \begin{bmatrix} 1 & 2 & 3 & 5 & 2 & 4 & 3 & 5 & 4 & 5 \end{bmatrix}$$

if `ISPAS` = `1` or

$$\texttt{IH} = \begin{bmatrix} 1 & 5 & 7 & 9 & 10 & 11 \end{bmatrix}, \qquad \texttt{JH} = \begin{bmatrix} 1 & 2 & 3 & 5 & 2 & 4 & 3 & 5 & 4 & 5 \end{bmatrix}$$

if `ISPAS` = `2`. In the first case, nonzero elements in the upper part of the Hessian matrix can be sorted in an arbitrary order (not only by rows as in the above example) and arrays `IH` and `JH` have to be declared with lengths $n + m$, where m is the number of nonzero elements. In the second case, nonzero elements can be sorted only by rows. Components of `IH` contain addresses of the diagonal elements in this sequence and components of `JH` contain corresponding column indices (note that `IH` has $n + 1$ elements and the last element is equal to $m + 1$). Arrays `IH` and `JH` have to be declared with lengths $n + 1$ and m, respectively.

Since the Hessian matrix can be indefinite, discrete versions of the Newton method are realized in the trust-region framework. Let B_k be a gradient-difference approximation of the Hessian matrix $G_k = G(x_k)$. Denote

$$Q_k(d) = \frac{1}{2} d^T B_k d + g_k^T d$$

the quadratic function which locally approximates the difference $F(x_k + d) - F(x_k)$,

$$\omega_k(d) = (B_k d + g_k)/\|g_k\|$$

the accuracy of the direction determination and

$$\rho_k(d) = \frac{F(x_k + d) - F(x_k)}{Q_k(d)}$$

the ratio of the actual and the predicted decrease of the objective function. Trust-region methods (used in subroutines PNED, PNEC, PGAD, PGAC) generate points $x_k \in R^n$, $k \in N$, in such a way that x_1 is arbitrary and

$$x_{k+1} = x_k + \alpha_k d_k, \quad k \in N, \tag{6}$$

where $d_k \in R^n$ are direction vectors and $\alpha_k \geq 0$ are step-sizes. Direction vectors $d_k \in R^n$ are chosen to satisfy conditions

$$\|d_k\| \leq \Delta_k, \tag{7}$$

$$\|d_k\| < \Delta_k \Rightarrow \|\omega_k(d_k)\| \leq \overline{\omega}_k, \tag{8}$$

$$-Q_k(d_k) \geq \underline{\sigma}\|g_k\| \min(\|d_k\|, \|g_k\|/\|B_k\|), \tag{9}$$

where $0 \leq \overline{\omega}_k \leq \overline{\omega} < 1$ and $0 < \underline{\sigma} < 1$ (we use value $\overline{\omega} = 0.9$ in our subroutines; $\underline{\sigma}$ is a theoretical value given implicitly). Step-sizes $\alpha_k \geq 0$ are selected so that

$$\rho_k(d_k) \leq 0 \Rightarrow \alpha_k = 0, \tag{10}$$

$$\rho_k(d_k) > 0 \Rightarrow \alpha_k = 1. \tag{11}$$

Trust-region radii $0 < \Delta_k \leq \overline{\Delta}$ are chosen in such a way that $0 < \Delta_1 \leq \overline{\Delta}$ (Δ_1 and $\overline{\Delta}$ are given by parameters XDEL and XMAX in our subroutines) and

$$\rho_k(d_k) < \underline{\rho} \Rightarrow \underline{\beta}\|d_k\| \leq \Delta_{k+1} \leq \overline{\beta}\|d_k\|, \tag{12}$$

$$\underline{\rho} \leq \rho_k(d_k) \leq \overline{\rho} \Rightarrow \Delta_{k+1} = \Delta_k, \tag{13}$$

$$\overline{\rho} < \rho_k(d_k) \Rightarrow \Delta_{k+1} = \min(\gamma\Delta_{k+1}, \overline{\Delta}), \tag{14}$$

where $0 < \underline{\beta} \leq \overline{\beta} < 1 < \gamma$ and $0 < \underline{\rho} < \overline{\rho} < 1$ (we use values $\underline{\beta} = 0.05$, $\overline{\beta} = 0.75$, $\gamma = 2$, $\underline{\rho} = 0.1$, $\overline{\rho} = 0.9$ in our subroutines). Note that the initial trust-region radius Δ_1 is computed by a simple formula when XDEL $= 0$. This formula depends on a gradient norm $\|g_k\|$ and contains a lower bound for F (parameter FMIN in our subroutines) if INITS $= 1$. If INITS $= 0$, parameter FMIN need not be defined.

The direction vector satisfying (7)–(9) can be computed by four different strategies. Subroutines PNED,PGAD are based on matrix decomposition methods. They use either the Dennis–Mei (double dog-leg) method [9](if MOS $=$ 1) or Moré–Sorensen method [25] (if MOS $=$ 2). Subroutines PNEC,PGAC are based on matrix iterative methods. They use either the Steihaug–Toint method [31], [32] (if MOS1 $=$ 1) or the shifted Steihaug–Toint method [16] (if MOS1 $=$ 2). To simplify the description of these methods, we omit the outer index k and denote the inner index by i.

3.1 Matrix decomposition Moré–Sorensen trust region method

The most sophisticated methods are based on a computation of the optimal locally constrained step. A vector $d \in R^n$ is obtained by solving a subproblem

$$\text{minimize} \quad Q(d) = \frac{1}{2} d^T B d + g^T d \quad \text{subject to} \quad \|d\| \le \Delta. \tag{15}$$

Necessary and sufficient conditions for this solution are

$$\|d\| \le \Delta, \quad (B + \lambda I)d + g = 0, \quad B + \lambda I \succeq 0, \quad \lambda \ge 0, \quad \lambda(\Delta - \|d\|) = 0 \tag{16}$$

(we use the symbol $\succeq$ for ordering by positive semidefiniteness). The Moré–Sorensen method [25] is based on solving a nonlinear equation

$$1/\|d(\lambda)\| = 1/\Delta \quad \text{with} \quad (B + \lambda I)d(\lambda) = -g$$

by the Newton method using a modification of the sparse Choleski decomposition of $B + \lambda I$ (we use the Gill–Murray decomposition [10]). More precisely, we determine $\underline{\mu}_1$ as the maximal diagonal element of the matrix $-B$, set $\underline{\lambda}_1 = \max(\underline{\mu}_1, 0)$, $\overline{\lambda}_1 = \|g\|/\Delta + \|B\|$, $\lambda_1 = \underline{\lambda}_1$ and for $i = 1, 2, 3, \ldots$ we proceed in the following way. Carry out the Gill–Murray decomposition $B + \lambda_i I + E_i = R_i^T R_i$. If $E_i \ne 0$, determine a vector $v_i \in R^n$ such that $\|v_i\| = 1$ and $v_i^T (B + \lambda_i I) v_i < 0$, set $\underline{\mu}_i = \lambda_i - v_i^T (B + \lambda_i I) v_i$, $\underline{\lambda}_i = \underline{\mu}_i$, $\lambda_i = \underline{\lambda}_i$ and repeat this process (i.e., carry out the new Gill–Murray decomposition $B + \lambda_i I + E_i = R_i^T R_i$). If $E_i = 0$, compute a vector $d_i \in R^n$ by solving the equation $R_i^T R_i d_i + g = 0$. If $\|d_i\| > \overline{\delta}\Delta$, set $\underline{\lambda}_{i+1} = \lambda_i$ and $\overline{\lambda}_{i+1} = \overline{\lambda}_i$. If $\underline{\delta}\Delta \le \|d_i\| \le \overline{\delta}\Delta$ or $\|d_i\| < \underline{\delta}\Delta$ and $\lambda_i = 0$, set $d = d_i$ and terminate the computation. If $\|d_i\| < \underline{\delta}\Delta$ and $\lambda_i \ne 0$, set $\underline{\lambda}_{i+1} = \underline{\lambda}_i$, $\overline{\lambda}_{i+1} = \lambda_i$, determine a vector $v_i \in R^n$ such that $\|v_i\| = 1$ and $v_i^T d_i \ge 0$, which is a good approximation of an eigenvector of the matrix B corresponding to its minimal eigenvalue, and compute a number $\alpha_i \ge 0$ such that $\|d_i + \alpha_i v_i\| = \Delta$. If

$$\alpha_i^2 \|R_i v_i\|^2 \le (1 - \underline{\delta}^2)(\|R_i d_i\|^2 + \lambda_i \Delta^2),$$

set $d = d_i + \alpha_i v_i$ and terminate the computation, otherwise set $\underline{\mu}_i = \lambda_i - \|R_i v_i\|^2$. In this case or if $\|d_i\| > \overline{\delta}\Delta$, compute a vector $v_i \in R^n$ by solving the equation $R_i^T v_i = d_i$ and set

$$\lambda_{i+1} := \lambda_i + \frac{\|d_i\|^2}{\|v_i\|^2} \left(\frac{\|d_i\| - \Delta}{\Delta} \right).$$

If $\lambda_{i+1} < \underline{\lambda}_{i+1}$, set $\lambda_{i+1} = \underline{\lambda}_{i+1}$. If $\lambda_{i+1} > \overline{\lambda}_{i+1}$, set $\lambda_{i+1} = \overline{\lambda}_{i+1}$. We use values $\underline{\delta} = 0.9$ and $\overline{\delta} = 1.1$ in our subroutines.

3.2 Matrix decomposition Dennis–Mei trust region method

The Moré–Sorensen method is very robust but requires 2-3 Choleski-type decompositions per iteration on average. Simpler methods are based on minimization of $Q(d)$ on a two-dimensional subspace containing the Cauchy step $d_C = -(g^T g / g^T B g) g$ and the Newton step $d_N = -B^{-1} g$. The Dennis–Mei double dog-leg method described in [9] seeks d as a linear combination of these two steps. This method uses vectors $d = d_N$ if $\|d_N\| \le \Delta$ or

$d = (\Delta/\|d_C\|)d_C$ if $\|d_C\| \geq \Delta$. In the remaining case (if $\|d_C\| < \Delta < \|d_N\|$), d is a convex combination of d_C and τd_N, where $\tau = \max(d_C^T d_C/d_C^T d_N, \Delta/\|d_N\|)$, such that $\|d\| = \Delta$.

The Newton step is computed by using the sparse Gill–Murray decomposition [10], which has the form $B + E = LDL^T = R^T R$, where E is a positive semidefinite diagonal matrix (equal to zero when B is positive definite), L is a lower triangular matrix, D is a positive definite diagonal matrix and R is an upper triangular matrix. The matrix $LDL^T = R^T R$ then replaces B in $Q(d)$. The Dennis–Mei method requires only one Choleski-type decomposition per iteration.

3.3 Matrix iterative Steihaug–Toint trust region method

If B is not sufficiently sparse, then the sparse Choleski-type decomposition of B is expensive. In this case, methods based on preconditioned conjugate gradient (CG) iterations are more suitable. Steihaug [31] and Toint [32] proposed a method based on the fact that $Q(d_{i+1}) < Q(d_i)$ and $\|d_{i+1}\|_C > \|d_i\|_C$ (where $\|d_i\|_C^2 = d_i^T C d_i$) hold in the preconditioned CG iterations if CG coefficients are positive. We either obtain an unconstrained solution with a sufficient precision or stop on the trust-region boundary if a negative curvature is indicated or if the trust-region is left. More precisely, we set $d_1 = 0$, $g_1 = g$, $p_1 = -C^{-1}g$ and for $i = 1, 2, 3, \ldots$ we proceed in the following way. If $\|g_i\| \leq \overline{\omega}\|g\|$, then set $d = d_i$ and terminate the computation, otherwise set

$$q_i = Bp_i, \qquad \alpha_i = g_i^T C^{-1} g_i / p_i^T q_i.$$

If $\alpha_i \leq 0$, determine $\alpha_i \geq 0$ in such a way that $\|d_i + \alpha_i p_i\| = \Delta$, set $d := d_i + \alpha_i p_i$ and terminate the computation, otherwise compute $d_{i+1} = d_i + \alpha_i p_i$. If $\|d_{i+1}\| \geq \Delta$, determine $\alpha_i \geq 0$ in such a way that $\|d_i + \alpha_i p_i\| = \Delta$, set $d := d_i + \alpha_i p_i$ and terminate the computation, otherwise compute

$$\begin{aligned} g_{i+1} &= g_i + \alpha_i q_i, \qquad \beta_i = g_{i+1}^T C^{-1} g_{i+1} / g_i^T C^{-1} g_i \\ p_{i+1} &= -C^{-1} g_{i+1} + \beta_i p_i. \end{aligned}$$

The matrix C serves as a preconditioner (symmetric and positive definite). If MOS2 $= 1$ (MOS2 is a parameter of subroutines PNEC,PGAC), then no preconditioning is performed ($C = I$), if MOS2 $= 2$, an incomplete Choleski decomposition of the matrix B is used, and if MOS2 $= 3$, a preliminary solution obtained by the incomplete Choleski decomposition can be accepted. In this case, we first compute $p_1 = -C^{-1}g$. If $\|Bp_1 + g\| \leq \overline{\omega}\|g\|$, we set $d = p_1$ and terminate the computation, otherwise we continue by CG iterations as above.

There are two possible ways that the Steihaug–Toint method can be preconditioned. The first way uses norms $\|d_i\|_{C_i}$ (instead of $\|d_i\|$) in (7)–(14), where C_i are preconditioners chosen. This possibility has been tested in [11] and showed that such a way is not always efficient. This is caused by the fact that norms $\|d_i\|_{C_i}$, $i \in N$, vary considerably in the major iterations and preconditioners C_i, $i \in N$, can be ill-conditioned. The second way uses Euclidean norms in (7)–(14) even if arbitrary preconditioners C_i, $i \in N$, are used. In this case the trust region can be left prematurely and the direction vector obtained can be farther from the optimal locally-constrained step than that obtained without preconditioning. This shortcoming is usually compensated by the rapid convergence of the preconditioned CG method. Our computational experiments indicated that the second way is more efficient in general and we use it in our subroutines.

3.4 Matrix iterative shifted Steihaug–Toint trust region method

Since the optimal locally constrained step has to satisfy the equation $(B + \lambda I)d + g = 0$, where $\lambda \geq 0$ is the optimal Lagrange multiplier (see (16)), it seems to be advantageous to apply the Steihaug–Toint method to the subproblem

$$\text{minimize} \quad \tilde{Q}(d) = Q_{\tilde{\lambda}}(d) = \frac{1}{2} d^T (B + \tilde{\lambda} I) d + g^T d \quad \text{s.t.} \quad \|d\| \leq \Delta, \tag{17}$$

where $\tilde{\lambda} \geq 0$ is an approximation of the optimal λ. The number $\tilde{\lambda} \geq 0$ is found by solving a small-size subproblem

$$\frac{1}{2} \tilde{d}^T T \tilde{d} + \|g\| e_1^T \tilde{d} \tag{18}$$

with the tridiagonal matrix T obtained by using a small number of Lanczos steps. This method combines good properties of the Moré–Sorensen and the Steihaug–Toint methods and can be successfully preconditioned by the second way described in the previous subsection. The point on the trust-region boundary obtained by this method is usually closer to the optimal solution in comparison with the point obtained by the original Steihaug–Toint method.

The above considerations form a basis for the shifted Steihaug–Toint method proposed in [16]. This method consists of the three steps:

1. Let m = `MOS1` (the default value is `MOS1` = 5). Determine a tridiagonal matrix T of order m by using m steps of the (unpreconditioned) Lanczos method (described, e.g., in [11], [14]) applied to the matrix B with the initial vector g.

2. Solve the subproblem

$$\text{minimize} \quad \frac{1}{2} \tilde{d}^T T \tilde{d} + \|g\| e_1^T \tilde{d} \quad \text{subject to} \quad \|\tilde{d}\| \leq \Delta \tag{19}$$

   by using the Moré–Sorensen method described in Section 3.1 to obtain a Lagrange multiplier $\tilde{\lambda}$.

3. Apply the (preconditioned) Steihaug–Toint method described in Section 3.3 to the subproblem

$$\text{minimize} \quad \frac{1}{2} d^T (B + \tilde{\lambda} I) d + g^T d \quad \text{subject to} \quad \|d\| \leq \Delta \tag{20}$$

   to obtain a direction vector $d = d(\tilde{\lambda})$.

Let $\tilde{\lambda}$ be the Lagrange multiplier of small-size subproblem (19) and λ be the Lagrange multiplier obtained by the Moré–Sorensen method applied to the original trust-region subproblem (15). It can be shown (see [16]) that $0 \leq \tilde{\lambda} \leq \lambda$. This inequality assures that $\lambda = 0$ implies $\tilde{\lambda} = 0$ so $\|d\| < \Delta$ implies $\tilde{\lambda} = 0$. Thus the shifted Steihaug–Toint method reduces to the standard one in this case. At the same time, if B is positive definite and $\tilde{\lambda} > 0$, then one has $\Delta \leq \|(B + \tilde{\lambda} I)^{-1} g\| < \|B^{-1} g\|$. Thus the unconstrained minimizer of the shifted quadratic function (20) is closer to the trust-region boundary than the unconstrained minimizer of the original quadratic function (15) and we can expect that $d(\tilde{\lambda})$ is closer to the optimal locally constrained step than $d(0)$. Finally, if $\tilde{\lambda} > 0$, then the matrix $B + \tilde{\lambda} I$ is better conditioned than B and we can expect that the shifted Steihaug–Toint method will converge more rapidly than the original one.

4 Methods for partially separable problems

Consider a function of the form

$$F(x) = \sum_{i=1}^{n_a} f_i(x), \tag{21}$$

where $f_i(x)$, $1 \le i \le n_a$ (n_a is usually large), are smooth particular functions depending on a small number of variables (n_i, say). In this case, the Jacobian matrix $J(x) = [J_{ij}(x)] = [\partial f_i(x)/\partial x_j]$ is sparse. In subroutines PSED, PSEC, and PSEN, the sparsity pattern of the Jacobian matrix is stored in the coordinate form (if $\texttt{ISPAS} = \texttt{1}$) or in the standard compressed row format (if $\texttt{ISPAS} = \texttt{2}$) using arrays IAG and JAG. For example, if the Jacobian matrix has the pattern

$$J = \begin{bmatrix} * & * & 0 & * \\ * & * & * & 0 \\ * & 0 & 0 & * \\ 0 & * & * & 0 \\ * & 0 & * & 0 \end{bmatrix}$$

(asterisks denote nonzero elements) then arrays IAG and JAG contain elements

$$\begin{aligned} \texttt{IAG} &= \begin{bmatrix} 1 & 1 & 1 & 2 & 2 & 2 & 3 & 3 & 4 & 4 & 5 & 5 \end{bmatrix}, \\ \texttt{JAG} &= \begin{bmatrix} 1 & 2 & 4 & 1 & 2 & 3 & 1 & 4 & 2 & 3 & 1 & 3 \end{bmatrix}, \end{aligned}$$

if $\texttt{ISPAS} = \texttt{1}$ or

$$\texttt{IAG} = \begin{bmatrix} 1 & 4 & 7 & 9 & 11 & 13 \end{bmatrix}, \qquad \texttt{JAG} = \begin{bmatrix} 1 & 2 & 4 & 1 & 2 & 3 & 1 & 4 & 2 & 3 & 1 & 3 \end{bmatrix},$$

if $\texttt{ISPAS} = \texttt{2}$. In the first case, nonzero elements can be sorted in an arbitrary order (not only by rows as in the above example). Arrays IAG and JAG have to be declared with lengths $n_a + m_a$ and m_a, respectively, where m_a is the number of nonzero elements. In the second case, nonzero elements can be sorted only by rows. Components of IAG contain total numbers of nonzero elements in all previous rows increased by 1 and elements of JAG contain corresponding column indices (note that IAG has $n_a + 1$ elements and the last element is equal to $m_a + 1$). Arrays IAG and JAG have to be declared with lengths $n_a + 1$ and m_a, respectively. This representation of sparse Jacobian matrices is also used in subroutines PGAD, PGAC, PMAX, PSUM, PEQN, PEQL described in the subsequent sections.

Using the sparsity pattern of the Jacobian matrix, we can define packed gradients $\hat{g}_i(x) \in R^{n_i}$ and packed Hessian matrices $\hat{G}_i(x) \in R^{n_i \times n_i}$ of functions $f_i(x)$, $1 \le i \le n_a$, as dense but small-size vectors and matrices. Note that $\hat{g}_i(x) = Z_i^T g_i(x)$, $\hat{G}_i(x) = Z_i^T G_i(x) Z_i$ and $g_i(x) = Z_i \hat{g}_i(x)$, $G_i(x) = Z_i \hat{G}_i(x) Z_i^T$, $1 \le i \le n_a$, where $g_i(x)$ and $G_i(x)$ are original gradients and Hessian matrices of functions $f_i(x)$, respectively, and $Z_i \in R^{n \times n_i}$ are matrices containing columns of the unit matrix corresponding to the variables appearing in $f_i(x)$.

Methods for partially separable problems are implemented in the line-search framework mentioned in Section 2. A direction vector d_k is computed by solving a system of linear equations with a matrix B_k, which is an approximation of the Hessian matrix computed from approximations of packed Hessian matrices, see (24). Subroutines PSED and PSEN

use the Gill–Murray matrix decomposition. In this case, B_k is replaced by $L_k D_k L_k^T = B_k + E_k$, where L_k is a lower triangular matrix, D_k is a positive definite diagonal matrix, and E_k is a positive semidefinite diagonal matrix chosen in such a way that $B_k + E_k$ is positive definite (more details are given in [10]). Subroutine PSEC uses the preconditioned conjugate gradient method described in Subsection 2.3, where multiplications by B_k are explicitly used instead of (4) and $\overline{\omega} = 0.9$ in (5).

4.1 Partitioned variable metric methods

Subroutines PSED, PSEC are based on partitioned variable metric updates [12], which consider each particular function separately. Thus approximations $\hat{B}_i$, $1 \le i \le n_a$, of the packed Hessian matrices $\hat{G}_i(x)$ are updated by using the quasi-Newton conditions $\hat{B}_i^+ \hat{s}_i = \hat{y}_i$, where $\hat{s}_i = Z_i^T s_i$ and $\hat{y}_i = \hat{g}_i^+ - \hat{g}_i$ (we omit outer index k and replace index $k+1$ by $+$ in this section). Therefore, a variable metric update can be used for each of the particular functions. However, there is a difference between the classic and the partitioned approach, since conditions $\hat{s}_i^T \hat{y}_i > 0$, which are necessary for positive definiteness of $\hat{B}_i^+$, are not guaranteed for all $1 \le i \le n_a$. This difficulty is unavoidable and an efficient algorithm has to handle this situation.

Subroutines PSED, PSEC use three strategies. If MET $= 1$, then the safeguarded partitioned BFGS updates

$$\begin{aligned} \hat{B}_i^+ &= \hat{B}_i + \frac{\hat{y}_i \hat{y}_i^T}{\hat{s}_i^T \hat{y}_i} - \frac{\hat{B}_i \hat{s}_i (\hat{B}_i \hat{s}_i)^T}{\hat{s}_i^T \hat{B}_i \hat{s}_i}, \quad && \hat{s}_i^T \hat{y}_i > 0, \\ \hat{B}_i^+ &= \hat{B}_i, && \hat{s}_i^T \hat{y}_i \le 0 \end{aligned} \tag{22}$$

are used. If MET $= 2$, then the BFGS updates are combined with the rank-one updates

$$\begin{aligned} \hat{B}_i^+ &= \hat{B}_i + \frac{(\hat{y}_i - \hat{B}_i \hat{s}_i)(\hat{y}_i - \hat{B}_i \hat{s}_i)^T}{\hat{s}_i^T (\hat{y}_i - \hat{B}_i \hat{s}_i)}, \quad && |\hat{s}_i^T (\hat{y}_i - \hat{B}_i \hat{s}_i)| \ge \varepsilon_M |\hat{s}_i^T \hat{B}_i \hat{s}_i|, \\ \hat{B}_i^+ &= \hat{B}_i, && |\hat{s}_i^T (\hat{y}_i - \hat{B}_i \hat{s}_i)| < \varepsilon_M |\hat{s}_i^T \hat{B}_i \hat{s}_i|, \end{aligned} \tag{23}$$

where ε_M is the machine precision. We use a strategy, which is based on the observation that (22) usually leads to the loss of convergence if too many particular functions have indefinite Hessian matrices. We start with the partitioned BFGS update (22). If $n_- \ge \theta n_a$, where n_- is a number of particular functions with a negative curvature and θ is a threshold value, then (23) is used for all particular functions in all subsequent iterations (we use value $\theta = 1/2$ in the subroutines PSED, PSEC). If MET $= 3$, then packed matrices $\hat{B}_i \approx \hat{G}_i(x)$ are computed by using gradient differences. This strategy is in fact the partitioned discrete Newton method.

A disadvantage of partitioned variable metric methods (MET $= 1$, MET $= 2$) is the fact that approximations of packed Hessian matrices need to be stored. Therefore, the number of stored elements can be much greater than the number of nonzero elements in the sparse Hessian matrix. Moreover, operations with packed Hessian matrices (decomposition, multiplication) are usually unsuitable (time consuming). Thus the sparse approximation of the Hessian matrix

$$B = \sum_{i=1}^{n_a} Z_i \hat{B}_i Z_i^T \tag{24}$$

(stored as in Section 3) is constructed in subroutines PSED, PSEC. Then a direction vector d, used in line search, is computed by solving the linear system $Bd = -g$. The partitioned Newton method (MET = 3) does not store packed matrices $\hat{B}_i$, $1 \le i \le n_a$, since they are immediately added to matrix (24).

4.2 Partitioned variable metric method for nonsmooth functions

Assume that functions $f_i(x)$, $1 \le i \le n_a$, appearing in (21), are nonsmooth, locally Lipschitz, and we are able to compute (Clarke) subgradients $g_i \in \partial f_i(x)$, $1 \le i \le n_a$, at any point $x \in R^n$. Then also $F(x)$ is locally Lipschitz and since locally Lipschitz function is differentiable almost everywhere by the Rademacher theorem, usually $\partial F(x) = \{\nabla F(x)\}$. A special feature of nonsmooth functions is the fact that the gradient $\nabla F(x)$ changes discontinuously and is not small in the neighborhood of a local minimum. Thus the standard optimization methods cannot be used efficiently.

The most commonly used approach for solving nonsmooth optimization problems is based on the bundle principle. In this case, values $F(x_k)$, $g(x_k) \in \partial F(x_k)$ at a single point x_k are replaced by a bundle of values $F_j = F(z_j)$, $g_j \in \partial F(z_j)$ obtained at trial points z_j, $j \in \mathcal{J}_k \subset \{1, \dots, k\}$. This bundle of values serves for defining a piecewise quadratic function (with a quadratic regularizing term), which is used for direction determination by solving a quadratic programming subproblem. Usually, the bundle contains many dense subgradients. Thus a dense quadratic programming subproblem with many constraints has to be solved, which is unsuitable in the large-scale cases. This disadvantage can be overcome by the bundle variable metric method described in [37], which uses a bundle with three subgradients at most for defining a quadratic programming subproblem. Subroutine PSEN is based on the partitioned bundle variable metric method described in [23], which combines ideas from [37] with partitioned variable metric updates.

Using aggregate subgradients $\tilde{g}_k$ and aggregated subgradient locality measures $\tilde{\beta}_k$ (where $\tilde{g}_1 = g_1$ and $\tilde{\beta}_1 = 0$ in the first iteration), the partitioned bundle variable metric method generates a sequence of basic points $\{x_k\} \subset R^n$ and a sequence of trial points $\{z_k\} \subset R^n$ such that

$$x_{k+1} = x_k + \alpha_k^L d_k, \quad z_{k+1} = x_k + \alpha_k^R d_k,$$

where $d_k = -(B_k^{-1} + \rho I)\tilde{g}_k$ is a direction vector and $\alpha_k^R > 0$, $\alpha_k^R \ge \alpha_k^L \ge 0$ are appropriately chosen step-sizes. At the same time, B_k is a matrix obtained by partitioned variable metric updates and ρ is a correction parameter (parameter ETA3 in subroutine PSEN). Step-sizes α_k^R and α_k^L are chosen by a special line-search procedure in such a way that either

$$F(x_k + \alpha_k^L d_k) \le F(x_k) - \varepsilon_L \alpha_L^R w_k$$

or

$$d_k^T g(x_k + \alpha_k^R d_k) \ge \gamma_{k+1} - \varepsilon_R w_k.$$

Here $0 < \varepsilon_L < 1/2$, $\varepsilon_L < \varepsilon_R < 1$ are suitable constants (we use values $\varepsilon_L = 10^{-4}$, $\varepsilon_R = 0.25$ in our subroutine), $g(x_k + \alpha_k^R d_k) \in \partial F(x_k + \alpha_k^R d_k)$, $w_k = -(\tilde{g}_k)^T d_k + 2\tilde{\beta}_k$ and

$$\gamma_{k+1} = \max\left(|F(x^k) - F(x_k + \alpha_k^R d_k) + \alpha_k^R d_k^T g(x_k + \alpha_k^R d_k)|, \gamma \|\alpha_k^R d_k\|^2\right)$$

where γ is a subgradient locality measure parameter (parameter ETA5 in subroutine PSEN). In the first case (descent step) we set $z_{k+1} = x_{k+1} = x_k + \alpha_k^L d_k$, $\beta_{k+1} = 0$ and substitute

$\tilde{\beta}_{k+1} = 0$, $\tilde{g}_{k+1} = g_{k+1} \in \partial F(z_{k+1})$. In the second case (null step) we set $z_{k+1} = x_k + \alpha_k^R d_k$, $x_{k+1} = x_k$, $\beta_{k+1} = \gamma_{k+1}$ and determine $\tilde{\beta}_{k+1}$, $\tilde{g}_{k+1}$ by the aggregation procedure.

The aggregation procedure is very simple. Denoting by l the lowest index satisfying $x_l = x_k$ (index of the iteration after the last descent step) and using the subgradients g_l, g_{k+1}, $\tilde{g}_k$ and the subgradient locality measures $\beta_l = 0$, β_{k+1}, $\tilde{\beta}_k$, we determine multipliers

$$\lambda_k^1 \geq 0, \quad \lambda_k^2 \geq 0, \quad \lambda_k^3 \geq 0, \quad \lambda_k^1 + \lambda_k^2 + \lambda_k^3 = 1,$$

which minimize the quadratic function

$$(\lambda^1 g_l + \lambda^2 g_{k+1} + \lambda^3 \tilde{g}_k)^T (B_k^{-1} + \rho I)(\lambda^1 g_l + \lambda^2 g_{k+1} + \lambda^3 \tilde{g}_k) + 2(\lambda^1 \beta_l + \lambda^2 \beta_{k+1} + \lambda^3 \tilde{\beta}_k),$$

and set

$$\tilde{g}_{k+1} = \lambda_k^1 g_l + \lambda_k^2 g_{k+1} + \lambda_k^3 \tilde{g}_k, \quad \tilde{\beta}_{k+1} = \lambda_k^1 \beta_l + \lambda_k^2 \beta_{k+1} + \lambda_k^3 \tilde{\beta}_k.$$

After obtaining points x_{k+1}, z_{k+1} and subgradient $g_{k+1} \in \partial F(z_{k+1})$, the matrix B_k is updated. To satisfy conditions for the global convergence, we use special symmetric rank-one updates after null steps. This guarantees that elements of B_k do not decrease. After descent steps, the safeguarded BFGS updates can be used. As the function $F(x)$ is partially separable, the matrix B_k can be expressed in the form (24) (we omit outer index k and replace index $k+1$ by $+$). Thus we set

$$\begin{aligned} \hat{B}_i^+ &= \hat{B}_i + \frac{\hat{y}_i \hat{y}_i^T}{\hat{s}_i^T \hat{y}_i} - \frac{\hat{B}_i \hat{s}_i (\hat{B}_i \hat{s}_i)^T}{\hat{s}_i^T \hat{B}_i \hat{s}_i}, \quad && \hat{s}_i^T \hat{y}_i > 0, \\ \hat{B}_i^+ &= \hat{B}_i, && \hat{s}_i^T \hat{y}_i \leq 0 \end{aligned}$$

after a descent step or

$$\begin{aligned} \hat{B}_i^+ &= \hat{B}_i + \frac{(\hat{y}_i - \hat{B}_i \hat{s}_i)(\hat{y}_i - \hat{B}_i \hat{s}_i)^T}{\hat{s}_i^T (\hat{y}_i - \hat{B}_i \hat{s}_i)}, \quad && \hat{s}_i^T (\hat{y}_i - \hat{B}_i \hat{s}_i) \geq \varepsilon_M \hat{s}_i^T \hat{B}_i \hat{s}_i, \\ \hat{B}_i^+ &= \hat{B}_i, && \hat{s}_i^T (\hat{y}_i - \hat{B}_i \hat{s}_i) < \varepsilon_M \hat{s}_i^T \hat{B}_i \hat{s}_i \end{aligned}$$

after a null step (ε_M is the machine precision). Here $\hat{s}_i = Z_i^T s_i$ and $\hat{y}_i = \hat{g}_i^+ - \hat{g}_i = Z_i^T (g_i^+ - g_i)$, where g_i^+ and g_i are subgradients computed at points z^+ and x, respectively (aggregated subgradients $\tilde{g}_i^+$ and $\tilde{g}_i$ are not used in variable metric updates).

Since the quadratic programming subproblem used in the aggregation procedure is very simple, initial step-size $\alpha = 1$ need not be a good choice in connection with its solution. Therefore we store and use a bundle of values $F_j = F(z_j)$, $g_j \in \partial F(z_j)$ obtained at trial points z_j, $j \in \mathcal{J}_k = \{k - m, \ldots, k\}$. Here m is a size of the bundle (parameter `MB` of subroutine `PSEN`). These values are used for the construction of a piecewise linear function which serves for determination of better initial step-size. More details are given in [23].

5 Hybrid methods for nonlinear least-squares

Consider a function of the form

$$F(x) = \frac{1}{2} \sum_{i=1}^{n_a} f_i^2(x) = \frac{1}{2} f^T(x) f(x) \tag{25}$$

(sum of squares), where $f_i(x)$, $1 \le i \le n_a$ (n_a is usually large), are smooth functions depending on a small number of variables (n_i, say). In this case, the Jacobian matrix $J(x) = [J_{ij}(x)] = [\partial f_i(x)/\partial x_j]$ is sparse. The sparsity pattern of the Jacobian matrix is stored using arrays `IAG` and `JAG` in the way described in Section 4.

Using the Jacobian matrix, we can express the gradient $g(x)$ and the Hessian matrix $G(x)$ in the form

$$\begin{aligned} g(x) &= \sum_{i=1}^{n_a} f_i(x) g_i(x) = J^T(x) f(x), \\ G(x) &= \sum_{i=1}^{n_a} \big(g_i(x) g_i^T(x) + f_i(x) G_i(x) \big) = J^T(x) J(x) + C(x) \end{aligned}$$

($G_i(x)$ are Hessian matrices of $f_i(x)$, $1 \le i \le n_a$). The well-known Gauss-Newton method uses the matrix $J^T(x)J(x)$ instead of the Hessian matrix $G(x) = J^T(x)J(x) + C(x)$ (i.e., it omits the second order information contained in $C(x)$). We assume that the matrix $J^T(x)J(x)$ is sparse (then also $C(x)$ is sparse).

The matrix $J^T(x)J(x)$ is frequently ill-conditioned (even singular) so that the Gauss-Newton method and its modifications require trust-region realizations. For computing a trust-region step, subroutine `PGAD` uses matrix decomposition methods and subroutine `PGAC` uses matrix iterative methods. These methods and their choices (using variables `MOS` and `MOS1`), are described in Section 3.

If the minimum value $F(x^*)$ is large (large residual problem), the Gauss-Newton method can be inefficient. Therefore, modifications that use the estimation of the second-order term have been developed. These modifications are based on the fact (proven in [1]) that $(F_k - F_{k+1})/F_k \to 1$ if $F_k \to 0$ Q-superlinearly and $(F_k - F_{k+1})/F_k \to 0$ if $F_k \to F^* > 0$. Thus we can use the following philosophy. Let x_{k+1} be a vector obtained by the trust-region strategy described in Section 3. If $x_{k+1} \ne x_k$, we compute $F_{k+1} = F(x_{k+1})$, $J_{k+1} = J(x_{k+1})$ and set

$$\begin{aligned} B_{k+1} &= J_{k+1}^T J_{k+1}, & F_k - F_{k+1} &> \underline{\vartheta} F_k, \\ B_{k+1} &= J_{k+1}^T J_{k+1} + C_{k+1}, & F_k - F_{k+1} &\le \underline{\vartheta} F_k, \end{aligned}$$

where C_{k+1} is an approximation of the second order term and $\underline{\vartheta}$ is a suitable value (parameter `ETA` in subroutines `PGAD` and `PGAC`).

Large-scale correction matrix C_{k+1} cannot be efficiently obtained by dense variable metric updates [1], which are frequently used for medium-size problems. Fortunately, simple corrections utilizing sparsity also increase the efficiency of the Gauss-Newton method. In subroutines `PGAD` and `PGAC` we have implemented three hybrid methods proposed in [15] (specified by the variable `MEC`), which are described in the subsequent subsections. To simplify the notation, we omit outer index k and replace index $k+1$ by $+$.

5.1 Gauss-Newton method with sparse variable metric corrections

If `MEC` = `1`, the sparse variable metric corrections (Marwil updates) are used. In the first iteration (or after a restart) we use the matrix $B = J^T J$. In the subsequent iterations we set

$$\begin{aligned} B^+ &= (J^+)^T J^+, & F - F^+ &> \underline{\vartheta} F, \\ B^+ &= \mathcal{P}_S \mathcal{P}_{QG}((J^+)^T J^+), & F - F^+ &\le \underline{\vartheta} F, \end{aligned}$$

where $\mathcal{P}_S$ realizes an orthogonal projection into the subspace of symmetric matrices of order n and $\mathcal{P}_{QG}$ realizes an orthogonal projection into the intersection of the subspace of matrices having the same sparsity pattern as $J^T J$ and the linear manifold of matrices satisfying the quasi-Newton condition $Ws = y$ with $s = x^+ - x$, $y = g^+ - g$. Thus

$$\mathcal{P}_S W = (W + W^T)/2,$$

$$\begin{aligned} (\mathcal{P}_G W)_{ij} &= W_{ij}, \quad (J^T J)_{ij} \neq 0, \\ (\mathcal{P}_G W)_{ij} &= 0, \qquad (J^T J)_{ij} = 0, \end{aligned}$$

for a given square matrix W, and

$$\mathcal{P}_{QG}((J^+)^T J^+) = \mathcal{P}_G((J^+)^T J^+ + us^T),$$

where $u \in R^n$ is a solution of the linear system $Du = y - (J^+)^T J^+ s$ with a diagonal matrix D such that

$$D_{ii} = \sum_{(J^T J)_{ij} \neq 0} (e_j^T s)^2$$

(e_j is the j-th column of the unit matrix).

5.2 Gauss-Newton method with the Newton corrections

If $\texttt{MEC} = 2$, the Newton corrections are used. In the first iteration (or after a restart) we use the matrix $B = J^T J$. In the subsequent iterations we set

$$\begin{aligned} B^+ &= (J^+)^T J^+, & F - F^+ > \underline{\vartheta} F, \\ B^+ &= (J^+)^T J^+ + \sum_{i=1}^{n_a} f_i^+ G_i^+, & F - F^+ \leq \underline{\vartheta} F, \end{aligned}$$

where G_i^+, $1 \leq i \leq n_a$, are approximations of Hessian matrices determined by using gradient differences at the point x^+.

5.3 Gauss-Newton method with partitioned variable metric corrections

If $\texttt{MEC} = 3$, the partitioned variable metric corrections (symmetric rank-one updates) are used. In the first iteration (or after a restart) we use the matrix $B = J^T J$. In the subsequent iterations we set

$$\begin{aligned} B^+ &= (J^+)^T J^+, & F - F^+ > \underline{\vartheta} F, \\ B^+ &= (J^+)^T J^+ + \sum_{i=1}^{n_a} f_i^+ Z_i \hat{B}_i^+ Z_i^T, & F - F^+ \leq \underline{\vartheta} F, \end{aligned}$$

where Z_i, $1 \leq i \leq n_a$, are matrices defined in Section 4 and $\hat{B}_i^+$, $1 \leq i \leq n_a$, are packed matrices updated using symmetric rank-one formulas

$$\begin{aligned} \hat{B}_i^+ &= \hat{B}_i + \frac{(\hat{y}_i - \hat{B}_i \hat{s}_i)(\hat{y}_i - \hat{B}_i \hat{s}_i)^T}{\hat{s}_i^T (\hat{y}_i - \hat{B}_i \hat{s}_i)}, & |\hat{s}_i^T (\hat{y}_i - \hat{B}_i \hat{s}_i)| \geq \varepsilon_M |\hat{s}_i^T \hat{B}_i \hat{s}_i|, \\ \hat{B}_i^+ &= \hat{B}_i, & |\hat{s}_i^T (\hat{y}_i - \hat{B}_i \hat{s}_i)| < \varepsilon_M |\hat{s}_i^T \hat{B}_i \hat{s}_i| \end{aligned}$$

(ε_M is the machine precision), where $\hat{B}_i = I$, $1 \le i \le n_a$, in the first iteration (or after a restart). A disadvantage of partitioned variable metric corrections is the fact that approximations of packed Hessian matrices need to be stored. Thus the choice MEC = 3 is usually less suitable than choices MEC = 1 and MEC = 2.

6 Primal interior point methods for minimax optimization

Consider a function of the form

$$F(x) = \max_{1 \le i \le n_a} f_i(x) \tag{26}$$

(pointwise maximum), where $f_i(x)$, $1 \le i \le n_a$ (n_a is usually large), are smooth functions depending on a small number of variables (n_i, say). In this case, the Jacobian matrix $J(x) = [J_{ij}(x)] = [\partial f_i(x)/\partial x_j]$ is sparse. The sparsity pattern of the Jacobian matrix is stored using arrays IAG and JAG in the way described in Section 4.

Primal interior point methods for minimax optimization, proposed in [17], are based on three basic ideas. First, minimization of F is equivalent to the nonlinear programming problem with $n+1$ variables $x \in R^n$, $z \in R$:

$$\text{minimize} \quad z \quad \text{subject to} \quad f_i(x) \le z, \quad 1 \le i \le n_a.$$

Secondly, this constrained problem is replaced by a sequence of unconstrained problems

$$\text{minimize} \quad B_\mu(x,z) = z - \mu \sum_{i=1}^{n_a} \log(z - f_i(x)),$$

where $z > F(x)$ and $\mu > 0$ (we assume that $\mu \to 0$ monotonically). Finally, the extra variable z is eliminated by solving the scalar nonlinear equation

$$\sum_{i=1}^{n_a} \frac{\mu}{z - f_i(x)} = 1, \tag{27}$$

which follows from the first-order necessary conditions for the barrier function $B_\mu(x,z)$ with fixed $x \in R^n$. The above equation has a unique root $z_\mu(x)$ such that $F(x) + \mu \le z_\mu(x) \le F(x) + n_a \mu$. This approach leads to inexact unconstrained minimizations of functions $B_\mu(x) = B_\mu(x, z_\mu(x))$ for suitable values of μ using the fact that

$$\nabla B_\mu(x) = g_\mu(x) = J^T(x) u_\mu(x)$$

and

$$\nabla^2 B_\mu(x) = G_\mu(x) + J^T(x) V_\mu(x) J(x) - \frac{J^T(x) V_\mu(x) e e^T V_\mu(x) J(x)}{e^T V_\mu(x) e},$$

where

$$u_\mu(x) = \begin{bmatrix} \mu/(z_\mu(x) - f_1(x)) \\ \dots \\ \mu/(z_\mu(x) - f_{n_a}(x)) \end{bmatrix}, \quad e = \begin{bmatrix} 1 \\ \dots \\ 1 \end{bmatrix},$$

$$G_\mu(x) = \sum_{i=1}^{n_a} e_i^T u_\mu(x) \nabla^2 f_i(x),$$

$$V_\mu(x) = \operatorname{diag}\left(\mu/(z_\mu(x) - f_1(x))^2, \ldots, \mu/(z_\mu(x) - f_{n_a}(x))^2\right).$$

The Hessian matrix $\nabla^2 B_\mu(x)$ of the barrier function $B_\mu(x)$ is positive definite if $G_\mu(x)$ (the Hessian matrix of the Lagrangian function) is positive definite.

Subroutine `PMAX` is based on a line search realization of the Newton-like methods. Thus $x^+ = x + \alpha d$, where $\nabla^2 B_\mu(x) d = -g_\mu(x)$ and α is a suitable step-size. In fact, we use an approximation of $\nabla^2 B_\mu(x)$, such that $G_\mu(x)$ is determined either by partitioned variable metric updates described in Section 4 (if `MED` = `1`) or by gradient differences as in [5] (if `MED` = `2`). In the second case, the matrix $G_\mu(x)$ is not positive definite in general so a restart strategy guaranteeing descent is used (more details are given in [17]). If `MED` = `1`, then we define reduced approximations of the Hessian matrices $\tilde{G}_i = Z_i^T G_i Z_i$, $1 \le i \le n_a$, as in Section 4. New reduced approximations of the Hessian matrices are computed by the formulas

$$\begin{aligned} \tilde{G}_i^+ &= \frac{1}{\tilde{\gamma}_i}\left(\tilde{G}_i - \frac{\tilde{G}_i \tilde{s}_i \tilde{s}_i^T \tilde{G}_i}{\tilde{s}_i^T \tilde{G}_i \tilde{s}_i}\right) + \frac{\tilde{y}_i \tilde{y}_i^T}{\tilde{s}_i^T \tilde{y}_i}, \quad \tilde{s}_i^T \tilde{y}_i > 0, \\ \tilde{G}_i^+ &= \tilde{G}_i, \qquad\qquad\qquad\qquad\qquad\quad \tilde{s}_i^T \tilde{y}_i \le 0, \end{aligned} \tag{28}$$

where

$$\tilde{s}_i = Z_i^T (x^+ - x), \quad \tilde{y}_i = Z_i^T (\nabla f_i(x^+) - \nabla f_i(x)), \quad 1 \le i \le n_a,$$

and where either $\tilde{\gamma}_i = 1$ or $\tilde{\gamma} = \tilde{s}_i^T \tilde{G}_i \tilde{s}_i / \tilde{s}_i^T \tilde{y}_i$. The particular choice of $\tilde{\gamma}_i$ is determined by the controlled scaling strategy described in [19]. In the first iteration we set $\tilde{G}_i = I$, $1 \le i \le n_a$, where I are unit matrices of suitable orders. Finally, $G_i^+ = Z_i \tilde{G}_i^+ Z_i^T$, $1 \le i \le n_a$.

A very important part of the primal interior point method is an update of the barrier parameter μ. There are two requirements, which play opposite roles. First, $\mu \to 0$ should hold, since this is the main property of every interior-point method. On the other hand, round-off errors can cause that $z_\mu(x) = F(x)$ when μ is too small and $|F(x)|$ is sufficiently large (since $F(x) + \mu \le z_\mu(x) \le F(x) + n_a \mu$), which leads to a breakdown (division by $z_\mu(x) - F(x) = 0$). Thus a lower bound $\underline{\mu}$ for the barrier parameter (parameter `ETA5` in subroutine `PMAX`) is used.

The efficiency of the primal interior point method is also sensitive to the way in which the barrier parameter decreases. Subroutine `PMAX` uses the formula

$$\mu_{k+1} = \max\left(\tilde{\mu}_{k+1}, \, \underline{\mu}, \, 10\, \varepsilon_M |F(x_{k+1})|\right),$$

where $F(x_{k+1}) = \max_{1 \le i \le m} f_i(x_{k+1})$, ε_M is the machine precision, and

$$\tilde{\mu}_{k+1} = \min\left[\max(\lambda \mu_k, \, \mu_k/(100\mu_k + 1)), \, \max(\|g_{\mu_k}(x_k)\|^2, \, 10^{-2k})\right],$$

where λ is the rate of the barrier parameter decrease (parameter `ETA4` in subroutine `PMAX`).

Subroutine `PMAX` serves for minimization of three particular functions. If `IEXT` $<$ `0`, then function (26) is considered. If `IEXT` = `0`, then

$$F(x) = \max_{1 \le i \le n_a} |f_i(x)| = \max_{1 \le i \le n_a} \left[\max(f_i(x), -f_i(x))\right].$$

If `IEXT` $>$ `0`, then

$$F(x) = \max_{1 \le i \le n_a} (-f_i(x)).$$

7 Primal interior point methods for l_1 optimization

Consider a function of the form

$$F(x) = \sum_{i=1}^{n_a} |f_i(x)| \tag{29}$$

(a sum of absolute values), where $f_i(x)$, $1 \le i \le n_a$ (n_a is usually large), are smooth functions depending on a small number of variables (n_i, say). In this case, the Jacobian matrix $J(x) = [J_{ij}(x)] = [\partial f_i(x)/\partial x_j]$ is sparse. The sparsity pattern of the Jacobian matrix is stored using arrays `IAG` and `JAG` in the way described in Section 4.

Primal interior point methods for l_1 optimization, proposed in [18], are based on three basic ideas. First, minimization of F is equivalent to the nonlinear programming problem with $n + n_a$ variables $x \in R^n$, $z \in R^{n_a}$:

$$\text{minimize} \quad \sum_{i=1}^{n_a} z_i \quad \text{subject to} \quad -z_i \le f_i(x) \le z_i, \quad 1 \le i \le n_a.$$

Secondly, this constrained problem is replaced by a sequence of unconstrained problems

$$\begin{aligned} \text{minimize} \quad B_\mu(x,z) &= \sum_{i=1}^{n_a} z_i - \mu \sum_{i=1}^{n_a} \log(z_i - f_i(x)) - \mu \sum_{i=1}^{n_a} \log(z_i + f_i(x)) \\ &= \sum_{i=1}^{n_a} z_i - \mu \sum_{i=1}^{n_a} \log(z_i^2 - f_i^2(x)) \end{aligned}$$

where $z_i > |f_i(x)|$, $1 \le i \le n_a$, and $\mu > 0$ (we assume that $\mu \to 0$ monotonically). Finally, the extra variables z_i, $1 \le i \le n_a$, are eliminated by solving the set of quadratic equations

$$\frac{2\mu z_i}{z_i^2 - f_i^2(x)} = 1, \quad 1 \le i \le n_a,$$

which follow from the first-order necessary conditions for the barrier function $B_\mu(x,z)$ with fixed $x \in R^n$. Solutions of the above equations define a vector $z_\mu(x) \in R^{n_a}$, where

$$z_\mu(x)_i = e_i^T z_\mu(x) = \mu + \sqrt{\mu^2 + f_i^2(x)}, \quad 1 \le i \le n_a. \tag{30}$$

This approach leads to inexact unconstrained minimizations of functions $B_\mu(x) = B_\mu(x, z_\mu(x))$ for suitable values of μ using the fact that

$$\nabla B_\mu(x) = g_\mu(x) = J^T(x) u_\mu(x)$$

and

$$\nabla^2 B_\mu(x) = G_\mu(x) + J^T(x) V_\mu(x) J(x),$$

where

$$u_\mu(x) = \begin{bmatrix} 2\mu f_1(x)/(z_\mu(x)_1^2 - f_1^2(x)) \\ \dots \\ 2\mu f_{n_a}(x)/(z_\mu(x)_{n_a}^2 - f_{n_a}^2(x)) \end{bmatrix},$$

$$G_\mu(x) = \sum_{i=1}^{n_a} e_i^T u_\mu(x) \nabla^2 f_i(x),$$
$$V_\mu(x) = \mathrm{diag}\left(2\mu/(z_\mu(x)_1^2 + f_1^2(x)), \ldots, 2\mu/(z_\mu(x)_{n_a}^2 + f_{n_a}^2(x))\right).$$

The Hessian matrix $\nabla^2 B_\mu(x)$ of the barrier function $B_\mu(x)$ is positive definite if $G_\mu(x)$ (the Hessian matrix of the Lagrangian function) is positive definite.

Subroutine `PSUM` is based on a trust-region realization of the Newton-like methods. The Dennis–Mei (dog-leg) method described in Section 3 is used for computation of the trust-region step d using the quadratic model

$$Q(d) = \frac{1}{2} d^T \nabla^2 B_\mu(x) d + g_\mu^T(x) d$$

(more details are given in [18]). In fact, we use an approximation of $\nabla^2 B_\mu(x)$, such that $G_\mu(x)$ is determined either by partitioned variable metric updates described in Section 4 (if $\texttt{MED} = 1$) or by gradient differences as in [5] (if $\texttt{MED} = 2$). If $\texttt{MED} = 1$, then we define reduced approximations of the Hessian matrices $\tilde{G}_i = Z_i^T G_i Z_i$, $1 \le i \le n_a$, as in Section 4. New reduced approximations of the Hessian matrices are computed by the formulas (28) described in Section 6.

A very important part of the primal interior point method is an update of the barrier parameter μ. There are two requirements, which play opposite roles. First, $\mu \to 0$ should hold, since this is the main property of every interior-point method. On the other hand, round-off errors can cause that $z_\mu(x)_i^2 = f_i^2(x)$ when μ is too small and $|f_i(x)|$ is sufficiently large (see (30)), which leads to a breakdown (division by $z_\mu(x)_i^2 - f_i^2(x) = 0$). Thus a lower bound $\underline{\mu}$ for the barrier parameter (parameter `ETA5` in subroutine `PSUM`) is used.

The efficiency of the primal interior point method is also sensitive to the way in which the barrier parameter decreases. Subroutine `PSUM` uses the formula

$$\mu_{k+1} = \max(\underline{\mu}, \|g_k(x_k)\|^2) \quad \text{if} \quad \rho(d_k) \ge \underline{\rho} \quad \text{and} \quad \|g_k(x_k)\|^2 \le \mu_k/100,$$

and $\mu_{k+1} = \mu_k$ otherwise ($\rho(d_k)$ and $\underline{\rho}$ are defined in Section 3).

8 Methods for sparse systems of nonlinear equations

Consider the system of nonlinear equations

$$f(x) = 0, \tag{31}$$

where $f : R^n \to R^n$ is a continuously differentiable mapping and assume that the Jacobian matrix $J(x) = [J_{ij}(x)] = [\partial f_i(x)/\partial x_j]$ is sparse. The sparsity pattern of the Jacobian matrix is stored using arrays `IAG` and `JAG` in the way described in Section 4 (where $n_a = n$). Let A be an approximation of the Jacobian matrix $J = J(x)$ and let $F = F(x) = (1/2)\|f(x)\|^2$. Methods considered in this section are realized in the line-search framework. They generate a sequence of points $x_i \in R^n$, $i \in N$, such that

$$x_{k+1} = x_k + \alpha_k d_k, \quad k \in N, \tag{32}$$

where $d_k \in R^n$ is a direction vector determined as an approximate solution of the linear system $A_k d + f_k = 0$ such that

$$\|A_k d_k + f_k\| \le \overline{\omega}_k \|f_k\| \tag{33}$$

with the precision $0 \le \overline{\omega}_k \le \overline{\omega} < 1$ and α_k is the step-size chosen in such a way that it is the first member of the sequence α_k^j, $j \in N$, where $\alpha_k^1 = 1$ and $\underline{\beta}\alpha_k^j \le \alpha_k^{j+1} \le \overline{\beta}\alpha_k^j$ with $0 < \underline{\beta} \le \overline{\beta} < 1$, satisfying

$$F_{k+1} - F_k \le -\varepsilon_1 \alpha_k f_k^T A_k d_k, \tag{34}$$

with the line search parameter $0 < \varepsilon_1 < 1/2$. We use the values $\underline{\beta} = 0.1$, $\overline{\beta} = 0.9$ and $\varepsilon_1 = 10^{-4}$ in our subroutines. The value α_k^{j+1} can be determined by a bisection (`MES` $= 1$) or by a two-point quadratic interpolation (`MES` $= 2$) or by a three-point quadratic interpolation (`MES` $= 3$) or by a three-point cubic interpolation (`MES` $= 4$).

To obtain a superlinear rate of convergence, the condition $\overline{\omega}_k \to 0$ has to be satisfied. Therefore, we set

$$\overline{\omega}_k = \min(\max(\|f_k\|^\nu, \gamma(\|f_k\|/\|f_{k-1}\|)^\alpha), 1/k, \overline{\omega}),$$

where $\nu = 1/2$, $\gamma = 1$, $\alpha = (1 + \sqrt{5})/2$ and $\overline{\omega} = 1/2$.

If $A_k \ne J_k$, then a safeguard based on restarts is used. It consists in setting $A_{k+1} = J_{k+1}$ if $j > \underline{j}$ or $A_k = J_k$ (with repeating the k-th iteration) if $j > \overline{j}$, where $0 < \underline{j} < \overline{j}$. We use the values $\underline{j} = 1$ and $\overline{j} = 5$. The restart of the form $A_k = J_k$ is also used whenever

$$-d_k^T J_k^T f_k \le \underline{\varepsilon} \|d_k\| \|J_k^T f_k\|,$$

where $0 < \underline{\varepsilon} < 1$ is a restart tolerance (we use the value $\underline{\varepsilon} = 10^{-12}$ in our subroutines).

The direction vector d_k (an approximate solution of the linear system $A_k d + f_k = 0$) is determined by using the preconditioned smoothed CGS method described in [35]. To simplify the description of this method, we omit the outer index k and denote the inner index by i. Let $h = A^T f$. We set $s_1 = 0$, $\overline{s}_1 = 0$, $r_1 = f$, $\overline{r}_1 = f$, $p_1 = f$, $u_1 = f$ and for $i = 1, 2, 3, \ldots$ we proceed in the following way. If $\|r_i\| \le \overline{\omega}\|f\|$, then set $d = s_i$ and terminate the process. Otherwise compute

$$\begin{aligned}
v_i &= AC^{-1}p_i, \quad \alpha_i = h^T \overline{r}_i / h^T v_i, \\
q_i &= u_i - \alpha_i v_i, \\
\overline{s}_{i+1} &= \overline{s}_i + \alpha_i C^{-1}(u_i + q_i), \\
\overline{r}_{i+1} &= \overline{r}_i + \alpha_i AC^{-1}(u_i + q_i), \quad \beta_i = h^T \overline{r}_{i+1} / h^T \overline{r}_i, \\
u_{i+1} &= \overline{r}_{i+1} + \beta_i q_i, \\
p_{i+1} &= u_{i+1} + \beta_i (q_i + \beta_i p_i), \\
[\lambda_i, \mu_i]^T &= \arg \min_{[\lambda,\mu]^T \in R^2} \|\overline{r}_{i+1} + \lambda(r_i - \overline{r}_{i+1}) + \mu v_i\|, \\
s_{i+1} &= \overline{s}_{i+1} + \lambda_i (s_i - \overline{s}_{i+1}) + \mu_i C^{-1} p_i, \\
r_{i+1} &= \overline{r}_{i+1} + \lambda_i (r_i - \overline{r}_{i+1}) + \mu_i v_i.
\end{aligned}$$

The matrix C serves as a preconditioner. The choice $C = I$ is used if `MOS2` $= 1$ or C is defined as an incomplete LU decomposition of the matrix A if `MOS2` $= 2$ (`MOS2` is a parameter of the subroutines `PEQN` and `PEQL`). If `MOS2` $= 3$, a preliminary solution obtained by the incomplete LU decomposition can be accepted. In this case, we first compute vectors $d_1 = -C^{-1}f$, $r_1 = Ad_1 + f$. If $\|r_1\| \le \overline{\omega}\|f\|$, then we set $d = d_1$ and terminate the process, otherwise we continue by CGS iterations as above.

More details concerning globally convergent line-search methods for systems of nonlinear equations can be found in [22].

8.1 Inexact discrete Newton method

Subroutine PEQN is an implementation of the inexact discrete Newton method. This simple method is based on the elementwise differentiation. We always set $A_k = J(x_k)$, where

$$J_{ij}(x) = \frac{f_i(x + \delta_j e_j) - f_i(x)}{\delta_j} \tag{35}$$

for all pairs (i, j) corresponding to structurally nonzero elements of $J(x)$. Thus we need m scalar function evaluations (i.e. m/n equivalent vector function evaluations), where m is the number of structurally nonzero elements of $J(x)$.

Nonzero elements of sparse Jacobian matrix $J(x)$ can be also derived by using the groupwise differentiation [4]. Since our comparative tests have shown that the efficiencies of both these approaches are practically the same (see [22]), we use the simpler elementwise differentiation in our subroutines.

8.2 Inverse column-update quasi-Newton method

Subroutine PEQL is an implementation of the inverse column update method, which is introduced in [24]. This method uses an approximation $S_k = A_k^{-1}$ of the inverse Jacobian matrix J_k^{-1} in (33). Therefore, we simply set $d_k = -S_k f_k$ instead of using the preconditioned smoothed CGS method if the restart is not used (if $A_k \neq J_k$). Denote by $s_k = x_{k+1} - x_k$, $s_{k-1} = x_k - x_{k-1}$, …, $s_{k-m} = x_{k-m+1} - x_{k-m}$ and $y_k = f_{k+1} - f_k$, $y_{k-1} = f_k - f_{k-1}$, …, $y_{k-m} = f_{k-m+1} - f_{k-m}$ the last m differences of points and function vectors, respectively, where the lower index $k - m$ corresponds to the iteration with the restart. Let $e_{k-1} = \arg\max_{e_i} |e_i^T y_{k-1}|$, …, $e_{k-m} = \arg\max_{e_i} |e_i^T y_{k-m}|$ (arg max is taken over all columns e_i, $1 \le i \le n$, of the unit matrix). Then the vector $S_k f_k$ can be computed by the formula

$$S_k f_k = S_{k-m} f_k + \frac{e_{k-1}^T f_k}{e_{k-1}^T y_{k-1}} v_{k-1} + \ldots + \frac{e_{k-m}^T f_k}{e_{k-m}^T y_{k-m}} v_{k-m},$$

where $v_{k-1} = d_{k-1} - S_{k-1} y_{k-1}$, …, $v_{k-m} = d_{k-m} - S_{k-m} y_{k-m}$ are vectors computed recursively by the formula

$$v_k = d_k - S_k y_k = d_k - S_{k-m} y_k - \frac{e_{k-1}^T y_k}{e_{k-1}^T y_{k-1}} v_{k-1} - \ldots - \frac{e_{k-m}^T y_k}{e_{k-m}^T y_{k-m}} v_{k-m}.$$

In both of these formulae we use the matrix $S_{k-m} = (L_{k-m} U_{k-m})^{-1}$, where $L_{k-m} U_{k-m}$ is the incomplete LU decomposition of the Jacobian matrix $J(x_{k-m})$ computed by (35). Note that the vectors e_{k-1}, …, e_{k-m} do not need to be stored. We only use indices of their unique nonzero elements. The limited memory column update method needs to be restarted periodically after $\overline{m}$ iterations (parameter MF in the subroutine PEQL), since at most $\overline{m}$ vectors can be stored.

9 Comparison of subroutines

In this section, we compare some of our subroutines with the most commonly known subroutines that solve the same problems. The comparison has been performed by using test collections TEST14 (with 1000 variables) for subroutines PLIS, PLIP, PNET, PNED, PNEC, PSED, PSEC, TEST15 (with 1000 variables) for subroutines PGAD, PGAC and TEST18 (with 3000 equations and unknowns) for subroutines PEQN, PEQL. These collections can be downloaded from www.cs.cas.cz/~luksan/test.html. Results of tests are listed in six tables. Rows corresponding to individual test problems contain the number of iterations NIT, the number of function evaluations NFV, the number of gradient evaluations NFG, the final value of the objective function F, the value of the termination criterion G, and the cause of termination ITERM. The last row of every table contains the total number of NIT, NFV, NFG, the total computational time in seconds and the number of problems successfully solved.

Table 2 contains the comparison of PLIS and PLIP with the subroutine L-BFGS [40]. Subroutine L-BFGS had to be slightly modified to eliminate overflows. Thus we have added statement STPMAX=XMAX/MXVNOR(N,S) (MXVNOR(N,S) is the Euclidean norm of N dimensional vector S) to the subroutine MCSRCH, realizing the Moré-Thuente line search, and used XMAX=1.0D1 for some problems. The value M=10 (corresponding to MF=10 in PLIS and PLIP) has been used in the subroutine L-BFGS.

Table 3 contains the comparison of PNET with the subroutine TNMIN from TNPACK [30]. As in the previous case, we have added statement DELH=MIN(DELH,XMAX/DNORM) to the subroutine OURHDP and statement STPMAX=XMAX/MXVNOR(N,S) to the subroutine MLINES. The default values of parameters in OPTLIST have been used in the subroutine TNMIN.

Table 4 contains the comparison of PSED and PSEC with the Harwell subroutine VE08AD [33]. The values DIFGRD=10^{-8}, FKNOWN='FALSE', RESTRT='FALSE', TESTGX='FALSE', HESDIF='FALSE', STEPL(1)=-1 and STMAX=XMAX have been used in the subroutine VE08AD (XMAX, which has the same meaning as in PSED, PSEC, was sometimes tuned).

Table 5 contains the comparison of PGAD and PGAC with the Harwell subroutine VE10AD [34]. The values DIFGRD=10^{-8}, FKNOWN='FALSE', RESTRT='FALSE', TESTGX='FALSE', HESDIF='FALSE', STEPL(1)=-1 and STMAX=XMAX have been used in the subroutine VE10AD (XMAX, which has the same meaning as in PGAD, PGAC, was sometimes tuned).

Table 6 contains the comparison of PEQN and PEQL (using only function values) with the subroutine NITSOL [28]. Since NITSOL does not contain an internal preconditioner, the less efficient unpreconditioned iterative method was used.

Table 7 contains the comparison of PEQN and PEQL (using function values and the first derivatives) with the subroutine NLEQ1S [27]. Subroutine NLEQ1S frequently required very large working arrays and a complete LU decomposition was sometimes very time-consuming.

The results introduced in Table 2–Table 7 demonstrate that PLIS and PLIP are comparable with L-BFGS. Other our subroutines tested are more efficient then their equivalents.

Method	TLISU			TLIPU			LBFGS		
Problem	NIT	NFV	NFG	NIT	NFV	NFG	NIT	NFV	NFG
1	4988	5554	5554	5383	5417	5417	4935	5821	5821
2	425	454	454	530	557	557	4048	4375	4375
3	74	78	78	125	128	128	88	96	96
4	103	112	112	109	114	114	113	120	120
5	24	26	26	26	27	27	23	24	24
6	30	31	31	35	36	36	36	38	38
7	38	43	43	36	41	41	29	50	50
8	29	33	33	33	36	36	32	55	55
9	13	16	16	15	18	18	11	15	15
10	1540	1582	1582	2003	2030	2030	1367	1403	1403
11	114	138	138	157	175	175	174	213	213
12	248	267	267	337	350	350	278	297	297
13	7	8	8	9	10	10	12	13	13
14	10	12	12	8	10	10	1	3	3
15	2830	2929	2929	1226	1256	1256	2773	2861	2861
16	196	210	210	237	246	246	229	240	240
17	1007	1032	1032	598	604	604	968	998	998
18	1449	1474	1474	989	998	998	1685	1732	1732
19	1393	1431	1431	1261	1272	1272	1703	1759	1759
20	2129	2191	2191	2045	2058	2058	2379	2433	2433
21	2120	2169	2169	2175	2196	2196	1945	1998	1998
22	1305	1346	1346	1261	1292	1292	1652	1706	1706
Σ	20072	21136	21136	18598	18871	18871	24481	26250	26250

Method	TLISU			TLIPU			LBFGS		
Problem	F	G	ITERM	F	G	ITERM	F	G	IFLAG
1	9.6E−15	E−06	4	6.0E−14	E−06	4	6.1E−13	E−04	0
2	14.9945	E−05	2	3.57277	E−05	2	276.253	E−04	0
3	6.6E−10	E−06	4	3.4E−13	E−06	4	8.1E−11	E−06	0
4	269.500	E−06	4	269.500	E−06	4	269.500	E−04	0
5	1.3E−12	E−06	4	7.1E−12	E−06	4	4.5E−11	E−04	0
6	2.2E−11	E−06	4	1.4E−11	E−06	4	1.3E−11	E−05	0
7	335.137	E−06	4	336.937	E−06	4	336.937	E−04	−1
8	761775.	E−03	2	761775.	E−02	2	761775.	E−03	−1
9	316.436	E−06	4	316.436	E−06	4	316.436	E−04	0
10	−124.630	E−04	2	−124.950	E−04	2	−131.810	E−04	0
11	10.7766	E−06	4	10.7766	E−06	4	10.7766	E−04	0
12	982.274	E−04	2	982.274	E−04	2	982.274	E−04	0
13	1.7E−13	E−06	4	2.3E−15	E−07	4	1.6E−20	E−09	0
14	1.3E−09	E−06	4	1.3E−09	E−06	4	1.3E−09	E−05	0
15	1.92402	E−06	4	1.92402	E−06	4	1.92402	E−04	−1
16	−427.404	E−05	2	−427.404	E−04	2	−427.404	E−05	0
17	−3.8E−02	E−06	4	−3.8E−02	E−06	4	−3.8E−02	E−05	0
18	−2.5E−02	E−06	4	−2.5E−02	E−06	4	−2.5E−02	E−05	0
19	59.5986	E−05	2	59.5986	E−05	2	59.5986	E−04	0
20	−1.00014	E−06	4	−1.00014	E−06	4	−1.00014	E−06	0
21	2.13867	E−06	4	2.13866	E−06	4	2.13866	E−04	0
22	1.00000	E−06	4	1.00000	E−06	4	1.00000	E−06	0
Σ	TIME = 8.90		22	TIME = 8.82		22	TIME = 9.34		22

Table 2: Comparison of TLISU, TLIPU and LBFGS

Table 3: Comparison of TNETU and TNMIN

Method	TNETU						TNMIN					
Problem	NIT	NFV	NFG	F	G	ITERM	NIT	NFV	NFG	F	G	INFORM
1	1481	1656	26037	1.2E−16	E−06	4	1756	2597	34032	4.1E−20	E−08	1
2	132	387	7945	1.5E−16	E−08	4	70	144	4166	7.0E−17	E−07	1
3	19	20	110	4.2E−10	E−06	4	19	20	145	5.2E−10	E−06	1
4	19	20	230	269.500	E−07	4	18	19	261	269.500	E−07	1
5	12	13	49	4.7E−12	E−06	4	11	12	60	1.0E−10	E−05	1
6	13	14	76	3.7E−12	E−06	4	12	13	99	2.8E−11	E−05	1
7	9	10	37	336.937	E−06	4	8	9	42	336.937	E−07	1
8	11	12	58	761775.	E−07	4	6	7	30	761775.	E−01	1
9	7	11	28	316.436	E−07	4	7	14	27	316.436	E−04	1
10	75	153	3213	−133.610	E−08	4	72	152	4936	−126.610	E−07	1
11	33	45	181	10.7766	E−07	4	30	41	188	10.7766	E−10	1
12	23	30	457	982.274	E−08	4	26	30	467	982.274	E−04	1
13	7	8	16	5.3E−16	E−07	4	7	8	17	8.9E−31	E−14	1
14	1	2	1005	1.2E−09	E−07	4	1	2	1002	1.2E−09	E−06	1
15	14	15	4033	1.92402	E−07	4	8	9	2765	1.92402	E−04	1
16	13	17	295	−427.404	E−08	4	13	18	473	−427.404	E−06	1
17	4	5	810	−3.8E−02	E−06	4	3	4	996	−3.8E−02	E−09	1
18	4	5	1146	−2.5E−02	E−06	4	3	4	1156	−2.5E−02	E−10	1
19	10	11	1986	59.5986	E−06	4	7	8	1532	59.5986	E−04	1
20	18	39	3051	−1.00014	E−07	4	14	35	4009	−1.00014	E−06	1
21	7	8	4901	2.13866	E−08	4	7	8	5610	2.13866	E−09	1
22	55	145	4760	1.00000	E−08	4	37	94	13588	1.00000	E−06	1
Σ	1967	2626	60424	TIME = 6.95		22	2135	3248	75601	TIME = 17.20		22

Method	TSEDU			TSECU			VE08AD		
Problem	NIT	NFV	NFG	NIT	NFV	NFG	NIT	NFV	NFG
1	2654	3627	3627	2606	3566	3566	4669	686	686
2	105	179	179	108	177	177	152	193	193
3	40	45	45	40	45	45	44	51	51
4	37	45	45	37	45	45	65	75	75
5	16	17	17	16	17	17	272	416	416
6	38	40	40	38	40	40	62	76	76
7	22	26	26	26	31	31	249	463	463
8	26	40	40	25	39	39	10	19	19
9	193	202	202	191	210	210	44	64	64
10	227	258	258	233	264	264	74	123	123
11	100	127	127	113	144	144	108	166	166
12	28	29	29	28	29	29	72	108	108
13	1	2	2	1	2	2	7	10	10
14	25	28	28	25	28	28	20	28	28
15	8	15	15	27	41	41	65	87	87
16	25	35	35	25	35	35	19	26	26
17	15	17	17	15	17	17	23	31	31
18	5	11	11	8	12	12	29	40	40
19	19	23	23	19	23	23	24	29	29
20	37	97	97	42	74	74	40	57	57
21	37	40	40	37	40	40	28	38	38
22	55	211	211	48	192	192	114	231	231
Σ	3713	5114	5114	3708	5071	5071	6190	3017	3017

Method	TSEDU			TSECU			VE08AD		
Problem	F	G	ITERM	F	G	ITERM	F	G	IFLAG
1	7.9E−17	E−06	3	6.5E−18	E−07	3	4.9E−17	E−06	1
2	83.3161	E−06	4	1111.36	E−05	2	5.0E−17	E−06	1
3	2.6E−13	E−06	4	2.7E−13	E−06	4	1.4E−13	E−06	1
4	269.500	E−06	4	269.500	E−06	4	269.500	E−06	1
5	1.1E−12	E−06	4	1.1E−12	E−06	4	1.1E−11	E−06	1
6	5.5E−12	E−06	4	5.5E−12	E−06	4	6.8E−13	E−06	1
7	335.253	E−06	4	335.253	E−06	4	316.783	E−07	1
8	761775.	E−04	2	761775.	E−03	2	761775.	E−05	29
9	316.436	E−05	2	316.436	E−05	2	316.436	E−06	1
10	−125.810	E−04	2	−121.692	E−04	2	−125.790	E−05	29
11	10.7766	E−06	4	10.7766	E−07	4	10.7766	E−07	1
12	982.274	E−06	4	982.274	E−06	4	982.274	E−06	1
13	0.00000	0.000	4	0.00000	0.000	3	6.5E−21	E−09	1
14	1.0E−09	E−06	4	1.0E−09	E−06	4	1.2E−09	E−06	1
15	1.92402	E−07	4	1.92402	E−07	4	1.92402	E−06	1
16	−427.404	E−06	4	−427.404	E−06	4	−427.404	E−06	1
17	−3.8E−02	E−06	4	−3.8E−02	E−06	4	−3.8E−02	E−07	1
18	−2.5E−02	E−07	4	−0.5E−02	E−11	4	−2.5E−02	E−06	1
19	59.5986	E−06	4	59.5986	E−06	4	59.5986	E−06	1
20	−1.00014	E−08	4	−1.00014	E−09	4	−1.00014	E−06	1
21	2.13866	E−06	4	2.13866	E−06	4	2.13866	E−06	1
22	1.00000	E−07	4	1.00000	E−08	4	1.00000	E−06	1
Σ	TIME = 4.27		22	TIME = 7.63		22	TIME = 19.40		22

Table 4: Comparison of TSEDU, TSECU and VE08AD

Method	TGADU			TGACU			VE10AD		
Problem	NIT	NFV	NFG	NIT	NFV	NFG	NIT	NFV	NFG
1	1377	1379	1379	1108	1110	1110	1456	180	180
2	41	46	46	624	640	649	348	509	509
3	11	12	14	11	12	14	17	18	18
4	13	16	21	11	13	17	23	24	24
5	4	5	7	4	5	7	6	7	7
6	6	7	13	6	7	13	7	8	8
7	10	12	23	17	40	29	8	9	9
8	21	26	24	22	25	25	96	267	267
9	15	16	36	13	15	38	48	50	50
10	12	18	21	129	147	176	9	10	10
11	2587	2593	2649	3010	3016	3012	3233	404	404
12	16	20	23	205	226	236	18	34	34
13	17	21	28	123	132	152	19	26	26
14	5	8	18	7	8	32	6	7	7
15	6	7	15	13	20	42	8	9	9
16	15	21	40	14	15	35	13	14	14
17	15	20	19	29	34	33	18	26	26
18	42	44	45	49	53	52	922	2397	2397
19	15	16	23	15	16	23	18	25	25
20	26	27	29	17	18	32	18	33	33
21	10	11	17	15	18	23	26	28	28
22	26	32	45	47	59	98	24	30	30
Σ	4290	4357	4535	5489	5629	5848	6341	4115	4115

Method	TGADU			TGACU			VE10AD		
Problem	F	G	ITERM	F	G	ITERM	F	G	IFLAG
1	7.0E−23	E−09	3	0.00000	E+00	3	2.6E-16	E−06	1
2	2.2E−17	E−06	3	66.4089	E−07	4	4.7E-16	E−07	1
3	1.4E−10	E−06	4	2.0E−10	E−06	4	1.3E-10	E−06	1
4	134.750	E−06	4	134.750	E−07	4	134.750	E−06	1
5	1.1E−11	E−06	4	1.2E−11	E−06	4	7.9E-19	E−08	1
6	7.4E−27	E−12	3	7.9E−27	E−12	3	2.3E-17	E−07	1
7	60734.9	E−07	4	60734.9	E−05	6	60734.9	E−06	1
8	2.5E−09	E−06	4	1.3E−08	E−06	4	1.1E-09	E−07	1
9	2216.46	E−10	4	2216.46	E−06	4	2216.46	E−05	29
10	191.511	E−07	4	191.511	E−07	4	191.511	E−06	1
11	6.5E−28	E−12	3	4.0E−25	E−11	3	1.0E-16	E−07	1
12	19264.6	E−10	4	22287.9	E−08	4	22287.9	E−08	1
13	131234.	E−08	4	131234.	E−09	4	131234.	E−07	1
14	108.518	E−08	4	108.518	E−07	4	108.518	E−07	1
15	18.1763	E−06	4	18.1763	E−05	2	18.1763	E−09	1
16	2.51110	E−06	4	2.51110	E−09	4	2.51110	E−06	1
17	2.6E−17	E−08	3	1.4E−10	E−06	4	1.2E-12	E−06	1
18	1.5E−25	E−10	3	1.2E−22	E−09	3	8.4E-14	E−05	1
19	3.5E−15	E−06	4	3.4E−14	E−06	4	8.3E-17	E−06	1
20	3.8E−11	E−07	4	3.4E−12	E−07	4	1.1E-17	E−08	1
21	647.829	E−11	4	647.696	E−06	4	647.696	E−05	29
22	4486.97	E−07	4	4486.97	E−08	4	4486.97	E−06	1
Σ	TIME = 4.56		22	TIME = 5.01		22	TIME = 27.31		22

Table 5: Comparison of TGADU, TGACU and VE10AD

Table 6: Comparison of TEQNU, TEQLU and NITSOL

Method	TEQNU					TEQLU					NITSOL				
Problem	NIT	NFV	NFG	F	ITERM	NIT	NFV	NFG	F	ITERM	NIT	NFV	NFG	F	ITERM
1	10	41	0	2.2E−23	3	30	64	0	3.3E−19	3	37	14095	0	1.1E−15	0
2	9	46	0	1.1E−23	3	17	57	0	7.2E−20	3	14	967	0	0.16900	5
3	3	19	0	3.3E−20	3	5	11	0	8.6E−17	3	4	9	0	0.00000	0
4	7	23	0	3.5E−18	3	11	19	0	1.2E−19	3	10	44	0	0.00000	0
5	12	63	0	1.2E−17	3	20	56	0	3.4E−17	3	9	2548	0	142.883	5
6	17	52	0	1.1E−17	3	22	31	0	1.7E−17	3	41	207	0	3.2E−14	6
7	13	41	0	3.4E−20	3	25	42	0	1.4E−21	3	183	570	0	0.00000	0
8	13	73	0	1.3E−26	3	21	60	0	5.0E−29	3	31	187	0	2.0E−15	6
9	13	99	0	4.3E−22	3	32	71	0	2.2E−22	3	22	136	0	8.9E−15	6
10	5	41	0	8.0E−26	3	9	24	0	2.0E−21	3	9	80	0	2.8E−15	6
11	12	37	0	1.9E−26	3	16	23	0	1.2E−22	3	14	37	0	0.00000	0
12	18	55	0	1.3E−17	3	23	40	0	8.6E−17	3	31	152	0	7.3E−17	0
13	18	39	0	1.1E−17	3	24	32	0	2.3E−17	3	55	198	0	1.4E−14	6
14	4	13	0	7.7E−21	3	8	13	0	6.0E−22	3	10	92	0	3.2E−15	6
15	5	36	0	1.8E−18	3	12	28	0	1.2E−18	3	8	96	0	4.6E−15	6
16	53	319	0	4.6E−18	3	22	78	0	9.8E−21	3	243	706	0	0.00000	0
17	14	48	0	4.5E−23	3	17	43	0	1.3E−21	3	112	444	0	6.0E−13	6
18	26	79	0	2.5E−21	3	46	61	0	2.2E−18	3	24	1925	0	37.8584	5
19	2	7	0	3.1E−22	3	2	5	0	7.0E−19	3	30	15000	0	–	3
20	13	43	0	4.3E−21	3	18	30	0	1.6E−17	3	24	15000	0	–	3
21	12	37	0	2.0E−21	3	25	34	0	2.3E−17	3	23	15000	0	–	3
22	7	50	0	2.0E−20	3	14	45	0	1.9E−18	3	12	3582	0	2.3E−02	5
23	29	262	0	3.9E−18	3	23	106	0	1.9E−19	3	20	15000	0	–	3
24	6	31	0	8.2E−24	3	20	53	0	7.4E−18	3	16	4187	0	8.3E−15	6
25	9	46	0	1.5E−24	3	29	50	0	2.1E−18	3	15	1053	0	8.7E−15	6
26	12	61	0	6.1E−18	3	36	67	0	1.3E−18	3	25	1326	0	1.3E−12	6
27	10	51	0	2.8E−21	3	40	75	0	6.6E−18	3	19	3434	0	8.4E−15	6
28	10	60	0	2.3E−17	3	27	83	0	4.6E−19	3	24	658	0	1.2E−14	6
29	4	53	0	1.2E−20	3	12	95	0	2.1E−17	3	15	15000	0	–	3
30	12	162	0	2.2E−22	3	18	145	0	7.4E−17	3	33	15000	0	–	3
Σ	378	1987	0	TIME = 3.81	30	624	1541	0	TIME = 3.22	30	1113	126733	0	TIME = 84.28	20

Table 7: Comparison of TEQNU, TEQLU and NLEQ1S

Method	TEQNU					TEQLU					NLEQ1S				
Problem	NIT	NFV	NFG	F	ITERM	NIT	NFV	NFG	F	ITERM	NIT	NFV	NFG	F	IERR
1	10	11	10	2.2E−23	3	30	43	7	3.3E−19	3	14	16	14	6.0E−12	0
2	9	10	9	1.1E−23	3	17	34	6	1.4E−17	3	6	8	6	1.55409	3
3	3	4	3	2.4E−20	3	5	6	1	8.6E−17	3	4	5	4	2.4E−14	0
4	6	15	6	4.1E−17	3	10	19	4	1.3E−17	3	7	10	7	0.00000	0
5	6	12	6	9.6E−18	3	9	15	3	8.4E−18	3	1	1	1	148.879	80
6	17	18	17	1.0E−17	3	22	23	4	1.6E−17	3	24	25	24	6.1E−13	5
7	13	15	13	3.2E−20	3	25	32	5	2.0E−21	3	44	73	44	7.7E−11	0
8	13	21	13	5.6E−27	3	21	32	7	1.5E−30	3	29	35	29	4.3E−10	0
9	13	21	13	4.0E−22	3	32	35	6	2.5E−22	3	25	26	25	9.4E−15	0
10	5	6	5	1.4E−26	3	9	10	2	2.0E−21	3	6	7	6	1.8E−14	0
11	12	13	12	0.00000	3	16	17	3	1.2E−22	3	13	14	13	0.00000	0
12	18	19	18	1.3E−17	3	23	30	5	9.9E−17	3	25	26	25	6.9E−13	5
13	21	24	21	9.2E−17	3	27	30	6	4.5E−17	3	1	1	1	30.8162	80
14	4	5	4	7.5E−21	3	8	9	2	6.1E−22	3	5	6	5	2.4E−14	0
15	5	6	5	1.8E−18	3	12	16	2	1.2E−18	3	6	7	6	3.7E−14	0
16	34	35	34	2.1E−21	3	22	46	16	9.8E−21	3	16	17	16	0.00000	0
17	14	20	14	1.1E−24	3	17	31	6	2.4E−21	3	16	18	16	5.2E−13	0
18	32	33	32	1.1E−17	3	37	39	6	3.6E−21	3	19	23	19	6.4E−11	0
19	2	3	2	3.8E−17	3	2	3	1	7.0E−19	3	4	5	4	8.0E−16	0
20	13	17	13	8.6E−24	3	18	22	4	1.6E−17	3	15	16	15	4.1E−12	0
21	12	13	12	2.0E−21	3	25	26	4	2.4E−17	3	17	18	17	1.3E−15	0
22	7	22	7	2.1E−20	3	14	29	4	2.0E−18	3	9	11	9	3.3E−14	0
23	10	11	10	1.7E−18	3	21	38	7	8.0E−21	3	5	7	5	1.29373	3
24	6	7	6	8.0E−24	3	20	33	5	7.3E−18	3	7	8	7	1.3E−14	0
25	9	10	9	1.5E−24	3	29	30	5	2.1E−18	3	5	6	5	1.9E−14	0
26	12	13	12	1.8E−20	3	41	42	6	9.8E−17	3	8	9	8	5.4E−11	0
27	10	11	10	1.1E−20	3	40	43	8	2.6E−18	3	9	10	9	3.2E−12	0
28	10	20	10	2.3E−17	3	27	47	9	4.6E−19	3	8	9	8	1.7E−14	0
29	7	8	7	6.3E−17	3	14	43	7	1.3E−17	3	14	15	14	3.6E−11	5
30	11	16	11	2.3E−17	3	23	40	9	9.8E−19	3	9	10	9	1.1E−14	0
Σ	344	439	344	TIME = 3.31	30	616	863	160	TIME = 3.50	30	371	442	371	TIME = 254.25	26

Appendix

A Description of subroutines

In this section we describe easy-to-use subroutines PLISU, PLISS, PLIPU, PLIPS, PNETU, PNETS, PNEDU, PNEDS, PNECU, PNECS, PSEDU, PSEDS, PSECU, PSECS, PSENU, PGADU, PGADS, PGACU, PGACS, PMAXU, PSUMU, PEQNU, PEQLU, which can be called from the user's program. In the description of formal parameters we introduce a type of the argument denoted by two letters. The first letter is either I for integer arguments or R for double-precision real arguments. The second letter specifies whether the argument must have a value defined on the entry to the subroutine (I), whether it is a value which will be returned (O), or both (U), or whether it is an auxiliary value (A). Beside the formal parameters, we use a COMMON /STAT/ block containing statistical information. This block, used in each subroutine, has the form

```
COMMON /STAT/ NRES,NDEC,NIN,NIT,NFV,NFG,NFH
```

whose elements have the following meanings:

Element	Type	Significance
NRES	IO	Number of restarts.
NDEC	IO	Number of matrix decompositions.
NIN	IO	Number of inner iterations (for solving linear systems).
NIT	IO	Number of iterations.
NFV	IO	Number of function evaluations.
NFG	IO	Number of gradient evaluations.
NFH	IO	Number of Hessian evaluations.

Easy-to-use subroutines are called by the following statements:

```
CALL PLISU(NF,X,IPAR,RPAR,F,GMAX,IPRNT,ITERM)
CALL PLISS(NF,X,IX,XL,XU,IPAR,RPAR,F,GMAX,IPRNT,ITERM)
CALL PLIPU(NF,X,IPAR,RPAR,F,GMAX,IPRNT,ITERM)
CALL PLIPS(NF,X,IX,XL,XU,IPAR,RPAR,F,GMAX,IPRNT,ITERM)
CALL PNETU(NF,X,IPAR,RPAR,F,GMAX,IPRNT,ITERM)
CALL PNETS(NF,X,IX,XL,XU,IPAR,RPAR,F,GMAX,IPRNT,ITERM)
CALL PNEDU(NF,MH,X,IH,JH,IPAR,RPAR,F,GMAX,ISPAS,IPRNT,ITERM)
CALL PNEDS(NF,MH,X,IX,XL,XU,IH,JH,IPAR,RPAR,F,GMAX,ISPAS,IPRNT,ITERM)
CALL PNECU(NF,MH,X,IH,JH,IPAR,RPAR,F,GMAX,ISPAS,IPRNT,ITERM)
CALL PNECS(NF,MH,X,IX,XL,XU,IH,JH,IPAR,RPAR,F,GMAX,ISPAS,IPRNT,ITERM)
CALL PSEDU(NF,NA,MA,X,AF,IAG,JAG,IPAR,RPAR,F,GMAX,ISPAS,IPRNT,ITERM)
CALL PSEDS(NF,NA,MA,X,IX,XL,XU,AF,IAG,JAG,IPAR,RPAR,F,GMAX,ISPAS,IPRNT,ITERM)
CALL PSECU(NF,NA,MA,X,AF,IAG,JAG,IPAR,RPAR,F,GMAX,ISPAS,IPRNT,ITERM)
CALL PSECS(NF,NA,MA,X,IX,XL,XU,AF,IAG,JAG,IPAR,RPAR,F,GMAX,ISPAS,IPRNT,ITERM)
CALL PSENU(NF,NA,MA,X,AF,IAG,JAG,IPAR,RPAR,F,GMAX,ISPAS,IPRNT,ITERM)
CALL PGADU(NF,NA,MA,X,AF,IAG,JAG,IPAR,RPAR,F,GMAX,IDER,ISPAS,IPRNT,ITERM)
```

```
CALL PGADS(NF,NA,MA,X,IX,XL,XU,AF,IAG,JAG,IPAR,RPAR,F,GMAX,IDER,ISPAS,IPRNT,ITERM)
CALL PGACU(NF,NA,MA,X,AF,IAG,JAG,IPAR,RPAR,F,GMAX,IDER,ISPAS,IPRNT,ITERM)
CALL PGACS(NF,NA,MA,X,IX,XL,XU,AF,IAG,JAG,IPAR,RPAR,F,GMAX,IDER,ISPAS,IPRNT,ITERM)
CALL PMAXU(NF,NA,MA,X,AF,IAG,JAG,IPAR,RPAR,F,GMAX,IEXT,ISPAS,IPRNT,ITERM)
CALL PSUMU(NF,NA,MA,X,AF,IAG,JAG,IPAR,RPAR,F,GMAX,ISPAS,IPRNT,ITERM)
CALL PEQNU(N,MA,X,AF,IAG,JAG,IPAR,RPAR,F,GMAX,IDER,ISPAS,IPRNT,ITERM)
CALL PEQLU(N,MA,X,AF,IAG,JAG,IPAR,RPAR,F,GMAX,IDER,ISPAS,IPRNT,ITERM)
```

Their arguments have the following meanings:

Argument	Type	Significance
NF	II	Number of variables of the objective function.
NA	II	Number of partial functions.
N	II	Number of nonlinear equations and unknowns.
MH	II	Number of nonzero elements in the upper part of the Hessian matrix. This parameter is used as input only if ISPAS = 1 (it defines dimensions of arrays IH and JH in this case).
MA	II	Number of nonzero elements in the Jacobian matrix. This parameter is used as input only if ISPAS = 1 (it defines dimensions of arrays IAG and JAG in this case).
X(NF)	RU	On input, vector with the initial estimate to the solution. On output, the approximation to the minimum.
IX(NF)	II	Vector containing the box constraint types (significant only if box constraints are considered):<br>IX(I) = 0: the variable X(I) is unbounded,<br>IX(I) = 1: the lower bound X(I) ≥ XL(I),<br>IX(I) = 2: the upper bound X(I) ≤ XU(I),<br>IX(I) = 3: the two-side bound XL(I) ≤ X(I) ≤ XU(I),<br>IX(I) = 5: the variable X(I) is fixed (given by its initial estimate).
XL(NF)	RI	Vector with lower bounds for variables (significant only if box constraints are considered).
XU(NF)	RI	Vector with upper bounds for variables (significant only if box constraints are considered).
IH(NIH)	IU	Row indices of the nonzero elements in the upper part of the Hessian matrix (NIH = NF + MH) if ISPAS = 1. Indices in array JH of the diagonal elements of the Hessian matrix (NIH = NF + 1) if ISPAS = 2.
JH(NJH)	IU	Column indices of the nonzero elements in the upper part of the Hessian matrix (NJH = NF + MH if ISPAS = 1 or NJH = MH if ISPAS = 2).
AF(NA)	RO	Vector which contains values of partial functions.
IAG(NIAG)	IU	Row indices of nonzero elements of the Jacobian matrix (NIAG = NA + MA) if ISPAS = 1. Indices in array JAG of the first elements in the rows of the Jacobian matrix (NIAG = NA + 1) if ISPAS = 2.
JAG(NJAG)	IU	Column indices of nonzero elements of the Jacobian matrix (NJAG = NA + MA if ISPAS = 1 or NJAG = MA if ISPAS = 2).
IPAR(7)	IU	Integer parameters (see Table 8).

RPAR(9)	RU	Real parameters (see Table 8).
F	RO	Value of the objective function at the solution X.
GMAX	RO	Maximum absolute value (l_∞ norm) of the gradient of the Lagrangian function.
IEXT	II	The type of minimax:<br>IEXT < 0: minimization of the maximum positive value,<br>IEXT $= 0$: minimization of the maximum absolute value,<br>IEXT > 0: minimization of the maximum negative value.
IDER	II	Degree of analytically computed derivatives (0 or 1).
ISPAS	II	Sparse structure of the Hessian or Jacobian matrix:<br>ISPAS $= 1$: the coordinate form is used,<br>ISPAS $= 2$: the standard row compressed format is used.
IPRNT	II	Print specification:<br>IPRNT $= 0$: print is suppressed,<br>IPRNT $= 1$: basic print of final results,<br>IPRNT $= -1$: extended print of final results,<br>IPRNT $= 2$: basic print of intermediate and final results,<br>IPRNT $= -2$: extended print of intermediate and final results.
ITERM	IO	Variable that indicates the cause of termination:<br>ITERM $= 1$: if $\|X - XO\|$ was less than or equal to TOLX in two subsequent iterations (XO is the vector of variables in the previous iteration),<br>ITERM $= 2$: if $\|F - FO\|$ was less than or equal to TOLF in two subsequent iterations (FO is the function value in the previous iteration),<br>ITERM $= 3$: if F is less than or equal to TOLB,<br>ITERM $= 4$: if GMAX is less than or equal to TOLG,<br>ITERM $= 6$: if termination criterion was not satisfied, but the solution is probably acceptable,<br>ITERM $= 11$: if NIT exceeded MIT,<br>ITERM $= 12$: if NFV exceeded MFV,<br>ITERM $= 13$: if NFG exceeded MFG,<br>ITERM < 0: if the method failed.

Integer and real parameters IPAR and RPAR need not be defined by the user who is not familiar with details of optimization methods. If we assign zeroes to these parameters, the suitable default values are assumed. An advanced user can change these default values by his knowledge-based options. The individual parameters, presented in Table 8, have the following meanings:

Argument	Type	Significance
MIT	II	Maximum number of iterations; the choice MIT $= 0$ causes that the default value (see Table 9) will be taken.
MFV	II	Maximum number of function evaluations; the choice MFV $= 0$ causes that the default value (see Table 9) will be taken.
MFG	II	Maximum number of gradient evaluations; the choice MFG $= 0$ causes that the default value (see Table 9) will be taken.

Parameter	PLIS	PLIP	PNET	PNED	PNEC	PSED	PSEC	PSEN	PGAD	PGAC	PMAX	PSUM	PEQN	PEQL
IPAR(1)	MIT	MIT	MIT	MIT	MIT	MIT	MIT	MIT	MIT	MIT	MIT	MIT	MIT	MIT
IPAR(2)	MFV	MFV	MFV	MFV	MFV	MFV	MFV	MFV	MFV	MFV	MFV	MFV	MFV	MFV
IPAR(3)	-	-	MFG	MFG	MFG	MFG	MFG	-	MFG	MFG	MFG	MFG	-	-
IPAR(4)	IEST	IEST	IEST	IEST	IEST	IEST	IEST	IEST	MEC	MEC	IEST	IEST	-	-
IPAR(5)	-	MET	MOS1	MOS	MOS1	MET	MET	-	MOS	MOS1	MED	MED	MOS1	MOS1
IPAR(6)	-	-	MOS2	-	MOS2	-	MOS2	MB	-	MOS2	-	-	MOS2	MOS2
IPAR(7)	MF	MF	MF	IFIL	IFIL	IFIL	IFIL	IFIL	IFIL	IFIL	IFIL	IFIL	-	MF
RPAR(1)	XMAX	XMAX	XMAX	XMAX	XMAX	XMAX	XMAX	XMAX	XMAX	XMAX	XMAX	XMAX	XMAX	XMAX
RPAR(2)	TOLX	TOLX	TOLX	TOLX	TOLX	TOLX	TOLX	TOLX	TOLX	TOLX	TOLX	TOLX	TOLX	TOLX
RPAR(3)	TOLF	TOLF	TOLF	TOLF	TOLF	TOLF	TOLF	TOLF	TOLF	TOLF	TOLF	TOLF	TOLF	TOLF
RPAR(4)	TOLB	TOLB	TOLB	TOLB	TOLB	TOLB	TOLB	TOLB	TOLB	TOLB	TOLB	TOLB	TOLB	TOLB
RPAR(5)	TOLG	TOLG	TOLG	TOLG	TOLG	TOLG	TOLG	TOLG	TOLG	TOLG	TOLG	TOLG	TOLG	TOLG
RPAR(6)	FMIN	FMIN	FMIN	FMIN	FMIN	FMIN	FMIN	FMIN	FMIN	FMIN	FMIN	FMIN	-	-
RPAR(7)	-	-	-	XDEL	XDEL	-	-	-	XDEL	XDEL	-	XDEL	-	-
RPAR(8)	-	-	-	-	-	-	-	ETA3	ETA	ETA	ETA4	-	ETA2	ETA2
RPAR(9)	-	-	-	-	-	-	-	ETA5	-	-	ETA5	ETA5	-	-

Table 8: Integer and real parameters

`IEST` II Estimation of the minimum function value for the line search:

- `IEST` = 0: estimation is not used,
- `IEST` = 1: lower bound `FMIN` is used as an estimation for the minimum function value.

`MEC` II Variable that determines the method of a second order correction:

- `MEC` = 1: correction by the Marwil sparse variable metric update,
- `MEC` = 2: correction by differences of gradients (discrete Newton correction),
- `MEC` = 3: correction by the Griewank–Toint partitioned variable metric update (symmetric rank-one).

The choice `MEC` = 0 causes that the default value `MEC` = 2 will be taken.

`MED` II Variable that specifies the method used:

- `MED` = 1: partitioned variable metric method,
- `MED` = 2: safeguarded discrete Newton method.

The choice `MED` = 0 causes that the default value `MED` = 1 will be taken.

`MET` II In `PLIP`: Variable that specifies the limited-memory method:

- `MET` = 1: rank-one method,
- `MET` = 2: rank-two method.

The choice `MET` = 0 causes that the default value `MET` = 2 will be taken.

In `PSED,PSEC`: Variable that specifies the variable metric update:

- `MET` = 1: safeguarded BFGS method,
- `MET` = 2: combination of the BFGS and the symmetric rank-one method,
- `MET` = 3: discrete Newton method.

The choice MET $= 0$ causes that the default value MET $= 2$ will be taken.

MOS	II	Method for computing the trust-region step:<br>MOS $= 1$: double dog-leg method of Dennis and Mei,<br>MOS $= 2$: method of More and Sorensen for obtaining optimum locally constrained step.<br>The choice MOS $= 0$ causes that the default value MOS $= 2$ will be taken.
MOS1	II	In PNET: Choice of restarts after constraint change:<br>MOS1 $= 1$: restarts are suppressed,<br>MOS1 $= 2$: restarts with steepest descent directions are used.<br>The choice MOS1 $= 0$ causes that the default value MOS1 $= 1$ will be taken.<br>In PNEC, PGAC: Method for computing trust-region step:<br>MOS1 $= 1$: Steihaug–Toint conjugate gradient method,<br>MOS1 $= 2$: shifted Steihaug–Toint method with five Lanczos steps,<br>MOS1 > 2: shifted Steihaug–Toint method with MOS1 Lanczos steps.<br>The choice MOS1 $= 0$ causes that the default value MOS1 $= 2$ will be taken.<br>In PEQN,PEQL: Variable that specifies the smoothing strategy for the CGS method:<br>MOS1 $= 1$: smoothing is not used,<br>MOS1 $= 2$: single smoothing strategy is used,<br>MOS1 $= 3$: double smoothing strategy is used.<br>The choice MOS1 $= 0$ causes that the default value MOS1 $= 3$ will be taken.
MOS2	II	Choice of preconditioning strategy:<br>MOS2 $= 1$: preconditioning is not used,<br>MOS2 $= 2$: preconditioning by the incomplete Gill-Murray decomposition (in PNET,PNEC,PSEC,PGAC) or incomplete LU decomposition in PEQN,PEQL),<br>MOS2 $= 3$: preconditioning as in case MOS2; the preliminary solution of the preconditioned system is accepted if it satisfies the termination criteria.<br>The choice MOS2 $= 0$ causes that the default values MOS2 $= 1$ in PNET, MOS2 $= 2$ in PNEC,PSEC,PGAC or MOS2 $= 3$ in PEQN,PEQL will be taken.
MB	II	Dimension of a bundle used in the line search; the choice MB $= 0$ causes that the default value MB $= 20$ will be taken.
MF	II	The number of limited-memory variable metric updates in every iteration; the choice MF $= 0$ causes that the default values MF $= 10$ in PLIS,PLIP,PNET or MF $= 6$ in PEQL will be taken.
IFIL	II	Variable that specifies a relative size of the space reserved for fill-in; the choice IFIL $= 0$ causes that the default value IFIL $= 1$ will be taken.
XMAX	RI	Maximum stepsize; the choice XMAX $= 0$ causes that the default value (see Table 9) will be taken.
TOLX	RI	Tolerance for the change of the coordinate vector X; the choice TOLX $= 0$ causes that the default value (see Table 9) will be taken.
TOLF	RI	Tolerance for the change of function values; the choice TOLF $= 0$ causes that the default value (see Table 9) will be taken.
TOLB	RI	Minimum acceptable function value; the choice TOLB $= 0$ causes that the default value (see Table 9) will be taken.

TOLG	RI	Tolerance for the Lagrangian function gradient; the choice TOLG = 0 causes that the default value (see Table 9) will be taken.
FMIN	RI	Lower bound for the minimum function value. This value is not used if IEST=0.
XDEL	RI	Trust region step-size; the choice XDEL = 0 causes that a suitable default value will be computed.
ETA	RI	Parameter for switch between the Gauss-Newton method and variable metric correction; the choice ETA = 0 causes that the default value ETA = $1.5 \cdot 10^{-4}$ will be taken.
ETA2	RI	Damping parameter for an incomplete LU preconditioner; the choice ETA2 = 0 causes that the default value ETA2 = 0 will be taken.
ETA3	RI	Correction parameter; the choice ETA3 = 0 causes that the default value ETA3 = 10^{-12} will be taken.
ETA4	RI	Coefficient for the barrier parameter decrease; the choice ETA4 = 0 causes that the default value ETA4 = 0.85 will be taken.
ETA5	RI	In PSEN: Parameter for subgradient locality measure; the choice ETA5 = 0 causes that the default value ETA5 = 10^{-12} will be taken.<br>In PMAX,PSUM: Minimum permitted value of the barrier parameter; the choice ETA5 = 0 causes that the default value ETA5 = 10^{-10} in PMAX and ETA5 = 10^{-8} in PSUM will be taken.

Value	PLIS PLIP	PNET	PNED PNEC	PSED PSEC	PSEN	PGAD PGAC	PMAX	PSUM	PEQL PEQN
MIT	9000	5000	5000	9000	20000	5000	10000	10000	1000
MFV	9000	5000	5000	9000	20000	5000	10000	10000	1000
MFG	9000	30000	10000	9000	20000	10000	20000	20000	10000
XMAX	10^{16}	10^{16}	10^{16}	10^{16}	10^{16}	10^{16}	10^{16}	10^{16}	10^{16}
TOLX	10^{-16}	10^{-16}	10^{-16}	10^{-16}	10^{-16}	10^{-16}	10^{-16}	10^{-16}	10^{-16}
TOLF	10^{-14}	10^{-14}	10^{-14}	10^{-14}	10^{-12}	10^{-14}	10^{-14}	10^{-12}	10^{-16}
TOLB	10^{-16}	10^{-16}	10^{-16}	10^{-16}	10^{-12}	10^{-16}	10^{-16}	10^{-12}	10^{-16}
TOLG	10^{-6}	10^{-6}	10^{-6}	10^{-6}	10^{-8}	10^{-6}	10^{-6}	10^{-6}	10^{-6}

Table 9: Default values

The subroutines PLISU,PLISS,PLIPU,PLIPS,PNETU,PNETS,PNEDU,PNEDS,PNECU,PNECS require the user supplied subroutines OBJ,DOBJ that define the objective function and its gradient and have the form

```
SUBROUTINE  OBJ(NF,X,F)
SUBROUTINE DOBJ(NF,X,G)
```

The subroutines PSEDU,PSEDS,PSECU,PSECS,PSENU,PGADU,PGADS,PGACU,PGACS,PMAXU,PSUMU, PEQNU,PEQLU require the user supplied subroutines FUN,DFUN that define particular functions and their gradients and have the form

```
SUBROUTINE  FUN(NF,KA,X,FA)
SUBROUTINE DFUN(NF,KA,X,GA)
```

If IDER = 0, the subroutine DFUN can be empty. The arguments of the user supplied subroutines have the following meanings:

Argument	Type	Significance
NF	II	Number of variables, equations and unknowns.
KA	II	Index of the partial function.
X(NF)	RI	An estimate to the solution.
F	RO	Value of the objective function at the point X.
FA	RO	Value of the KA-th partial function at the point X.
G(NF)	RO	Gradient of the objective function at the point X.
GA(NF)	RO	Gradient of the KA-th smooth partial function (or an arbitrary subgradient of the KA-th nonsmooth partial function) at the point X.

B Verification of subroutines

In this section we report the results obtained by using test programs TLISU, TLISS, TLIPU, TLIPS, TNETU, TNETS, TNEDU, TNEDS, TNECU, TNECS, TSEDU, TSEDS, TSECU, TSECS, TSENU, TGADU, TGADS, TGACU, TGACS, TMAXU, TSUMU, TEQNU, TEQLU, which serve for demonstration, verification and testing of subroutines PLISU, PLISS, PLIPU, PLIPS, PNETU, PNETS, PNEDU, PNEDS, PNECU, PNECS, PSEDU, PSEDS, PSECU, PSECS, PSENU, PGADU, PGADS, PGACU, PGACS, PMAXU, PSUMU, PEQNU, PEQLU. These results are listed in the following tables (rows corresponding to individual test problems contain the number of iterations NIT, the number of function evaluations NFV, the number of gradient evaluations NFG, the final value of the objective function F, the value of the termination criterion G, and the cause of termination ITERM). The last row of every table contains the total number of NIT, NFV, NFG, and, moreover, for programs TNECU, TNECS, TSECU, TSECS, TGACU, TGACS, TEQNU, TEQLU also the total number of conjugate gradient iterations NCG. The total computational time in seconds is included. All computations reported were performed on a Pentium PC computer, under the Windows XP system using the Digital Visual Fortran (Version 6) compiler, in double-precision arithmetic.

The above test programs are based on test collections TEST14, TEST15, TEST18 described in [21], which can be downloaded from www.cs.cas.cz/~luksan/test.html. The box constraints are assumed in the form $-1 \le x_i \le 1$, $1 \le i \le n$. Subroutines for nonlinear equations were tested by problems with 3000 equations and unknowns. Subroutines for nonsmooth unconstrained optimization were tested by problems with 200 variables. The remaining subroutines were tested by problems with 1000 variables.

Problem	NIT	NFV	NFG	F	G	ITERM
1	4988	5554	5554	0.963780013E−14	0.891E−06	4
2	425	454	454	14.9944763	0.773E−05	2
3	74	78	78	0.655101686E−09	0.539E−06	4
4	103	112	112	269.499543	0.899E−06	4
5	24	26	26	0.130639280E−11	0.671E−06	4
6	30	31	31	0.216102227E−10	0.946E−06	4
7	38	43	43	335.137433	0.730E−06	4
8	29	33	33	761774.954	0.432E−03	2
9	13	16	16	316.436141	0.369E−06	4
10	1540	1582	1582	−124.630000	0.124E−04	2
11	114	138	138	10.7765879	0.380E−06	4
12	248	267	267	982.273617	0.123E−04	2
13	7	8	8	0.165734137E−12	0.453E−06	4
14	10	12	12	0.128729169E−08	0.916E−06	4
15	2830	2929	2929	1.92401599	0.936E−06	4
16	196	210	210	−427.404476	0.991E−05	2
17	1007	1032	1032	−0.379921091E−01	0.876E−06	4
18	1449	1474	1474	−0.245741193E−01	0.862E−06	4
19	1393	1431	1431	59.5986241	0.259E−05	2
20	2129	2191	2191	−1.00013520	0.908E−06	4
21	2120	2169	2169	2.13866377	0.927E−06	4
22	1305	1346	1346	1.00000000	0.982E−06	4
Σ	20072	21136	21136		TIME = 8.90	

Table 10: Results obtained by program TLISU

Problem	NIT	NFV	NFG	F	G	ITERM
1	5055	5595	5595	0.00000000	0.000E+00	3
2	2016	2289	2289	3926.45961	0.304E−04	2
3	95	106	106	0.217616100E-12	0.780E−06	4
4	58	64	64	269.522686	0.124E−05	2
5	24	26	26	0.130639280E-11	0.671E−06	4
6	30	31	31	0.216102227E-10	0.946E−06	4
7	31	35	35	337.722479	0.776E−06	4
8	50	58	58	761925.725	0.257E−03	2
9	504	506	506	428.056916	0.940E−07	4
10	1152	1211	1211	−82.0207503	0.176E−04	2
11	13	23	23	96517.2947	0.126E−08	4
12	79	88	88	4994.21410	0.325E−06	4
13	7	8	8	0.165734137E-12	0.453E−06	4
14	10	12	12	0.128729169E-08	0.916E−06	4
15	2830	2929	2929	1.92401599	0.936E−06	4
16	176	184	184	−427.391653	0.348E−04	2
17	1007	1032	1032	−0.379921091E-01	0.876E−06	4
18	1449	1474	1474	−0.245741193E-01	0.862E−06	4
19	1150	1183	1183	1654.94525	0.908E−05	2
20	2211	2274	2274	−1.00013520	0.886E−06	4
21	1280	1303	1303	2.41354873	0.997E−06	4
22	1562	1598	1598	1.00000000	0.786E−06	4
Σ	20789	22029	22029		TIME = 11.37	

Table 11: Results obtained by program TLISS

Problem	NIT	NFV	NFG	F	G	ITERM
1	5383	5417	5417	0.601022658E−13	0.599E−06	4
2	530	557	557	3.57276719	0.124E−05	2
3	125	128	128	0.338270284E−12	0.518E−06	4
4	109	114	114	269.499543	0.669E−06	4
5	26	27	27	0.710072396E−11	0.951E−06	4
6	35	36	36	0.142942272E−10	0.737E−06	4
7	36	41	41	336.937181	0.956E−06	4
8	33	36	36	761774.954	0.192E−02	2
9	15	18	18	316.436141	0.264E−06	4
10	2003	2030	2030	−124.950000	0.116E−04	2
11	157	175	175	10.7765879	0.299E−06	4
12	337	350	350	982.273617	0.145E−04	2
13	9	10	10	0.230414406E−14	0.642E−07	4
14	8	10	10	0.128834241E−08	0.977E−06	4
15	1226	1256	1256	1.92401599	0.970E−06	4
16	237	246	246	−427.404476	0.501E−04	2
17	598	604	604	−0.379921091E−01	0.908E−06	4
18	989	998	998	−0.245741193E−01	0.975E−06	4
19	1261	1272	1272	59.5986241	0.410E−05	2
20	2045	2058	2058	−1.00013520	0.911E−06	4
21	2175	2196	2196	2.13866377	0.996E−06	4
22	1261	1292	1292	1.00000000	0.927E−06	4
Σ	18598	18871	18871		TIME = 8.82	

Table 12: Results obtained by program TLIPU

Problem	NIT	NFV	NFG	F	G	ITERM
1	5263	5321	5321	0.530131995E−13	0.370E−05	2
2	2293	2447	2447	3930.43962	0.251E−04	2
3	127	132	132	0.210550150E−12	0.437E−06	4
4	70	72	72	269.522686	0.794E−06	4
5	26	27	27	0.710072396E−11	0.951E−06	4
6	35	36	36	0.142942272E−10	0.737E−06	4
7	37	43	43	336.937181	0.133E−05	2
8	59	65	65	761925.725	0.399E−03	2
9	508	510	510	428.056916	0.776E−06	4
10	1253	1277	1277	−82.5400568	0.120E−04	2
11	13	19	19	96517.2947	0.150E−04	2
12	95	102	102	4994.21410	0.790E−04	2
13	9	10	10	0.230414406E−14	0.642E−07	4
14	8	10	10	0.128834241E−08	0.977E−06	4
15	1226	1256	1256	1.92401599	0.970E−06	4
16	227	228	228	−427.391653	0.952E−05	2
17	598	604	604	−0.379921091E−01	0.908E−06	4
18	989	998	998	−0.245741193E−01	0.975E−06	4
19	1367	1383	1383	1654.94525	0.105E−04	2
20	2274	2303	2303	−1.00013520	0.798E−06	4
21	1196	1211	1211	2.41354873	0.975E−06	4
22	1361	1381	1381	1.00000000	0.962E−06	4
Σ	19034	19435	19435		TIME = 9.42	

Table 13: Results obtained by program TLIPS

Problem	NIT	NFV	NFG	F	G	ITERM
1	1481	1656	26037	0.117631766E−15	0.354E−06	4
2	132	387	7945	0.153382199E−15	0.988E−08	4
3	19	20	110	0.421204156E−09	0.353E−06	4
4	19	20	230	269.499543	0.779E−07	4
5	12	13	49	0.465606821E−11	0.364E−06	4
6	13	14	76	0.366783327E−11	0.404E−06	4
7	9	10	37	336.937181	0.248E−06	4
8	11	12	58	761774.954	0.155E−07	4
9	7	11	28	316.436141	0.158E−07	4
10	75	153	3213	−133.610000	0.777E−08	4
11	33	45	181	10.7765879	0.414E−07	4
12	23	30	457	982.273617	0.591E−08	4
13	7	8	16	0.533593908E−15	0.327E−07	4
14	1	2	1005	0.120245125E−08	0.879E−07	4
15	14	15	4033	1.92401599	0.468E−07	4
16	13	17	295	−427.404476	0.800E−08	4
17	4	5	810	−0.379921091E−01	0.537E−06	4
18	4	5	1146	−0.245741193E−01	0.425E−06	4
19	10	11	1986	59.5986241	0.423E−06	4
20	18	39	3051	−1.00013520	0.712E−07	4
21	7	8	4901	2.13866377	0.120E−08	4
22	55	145	4760	1.00000000	0.206E−08	4
Σ	1967	2626	60424		TIME = 6.95	

Table 14: Results obtained by program TNETU

Problem	NIT	NFV	NFG	F	G	ITERM
1	1611	1793	28524	0.00000000	0.000E+00	3
2	259	259	4418	3930.43956	0.230E−07	4
3	17	18	98	0.158634811E−08	0.954E−06	4
4	12	13	105	269.522686	0.103E−07	4
5	12	13	49	0.465606821E−11	0.364E−06	4
6	13	14	76	0.366783327E−11	0.404E−06	4
7	9	10	37	336.937181	0.248E−06	4
8	40	41	248	761925.725	0.281E−06	4
9	553	555	2056	428.056916	0.850E−07	4
10	112	137	2109	−84.1426617	0.732E−06	4
11	7	8	17	96517.2947	0.112E−11	4
12	133	136	2689	4994.21410	0.180E−06	4
13	7	8	16	0.533593908E−15	0.327E−07	4
14	1	2	1005	0.120245125E−08	0.879E−07	4
15	14	15	4033	1.92401599	0.468E−07	4
16	12	13	294	−427.391653	0.594E−06	4
17	4	5	810	−0.379921091E−01	0.537E−06	4
18	4	5	1146	−0.245741193E−01	0.425E−06	4
19	8	9	1902	1654.94525	0.690E−07	4
20	16	25	3254	−1.00013520	0.836E−08	4
21	4	5	1211	2.41354873	0.135E−06	4
22	52	137	4843	1.00000000	0.657E−06	4
Σ	2900	3221	58940		TIME = 8.56	

Table 15: Results obtained by program TNETS

Problem	NIT	NFV	NFG	F	G	ITERM
1	1421	1425	5688	0.465831486E−25	0.418E−12	3
2	39	45	200	0.231406390E−14	0.350E−06	4
3	17	18	108	0.839782900E−09	0.933E−06	4
4	24	25	100	269.499543	0.666E−10	4
5	11	12	72	0.795109456E−10	0.473E−06	4
6	13	16	196	0.125944855E−10	0.815E−06	4
7	12	13	78	336.937181	0.300E−06	4
8	4	5	90	761774.954	0.216E−06	4
9	7	9	16	316.436141	0.146E−06	4
10	69	75	630	−135.290000	0.291E−11	4
11	67	68	408	10.7765879	0.199E−06	4
12	127	128	512	982.273617	0.495E−09	4
13	6	7	28	0.598998674E−10	0.693E−06	4
14	2	3	18	0.129013604E−08	0.792E−06	4
15	9	10	40	1.92401599	0.414E−06	4
16	7	8	48	−427.404476	0.565E−07	4
17	8	9	54	−0.379921091E−01	0.314E−10	4
18	7	8	48	−0.245741193E−01	0.218E−09	4
19	6	7	42	59.5986241	0.952E−08	4
20	14	15	90	−1.00013520	0.139E−08	4
21	11	12	72	2.13866377	0.331E−08	4
22	30	34	186	1.00000000	0.164E−08	4
Σ	1911	1952	8724		TIME = 3.00	

Table 16: Results obtained by program TNEDU

Problem	NIT	NFV	NFG	F	G	ITERM
1	1420	1424	5680	0.00000000	0.000E+00	3
2	128	130	640	1980.05047	0.911E−10	4
3	17	19	108	0.189355864E−09	0.340E−06	4
4	10	12	44	269.522686	0.328E−09	4
5	13	15	84	0.391905635E−12	0.536E−06	4
6	13	14	196	0.136396633E−11	0.901E−06	4
7	30	32	186	336.920046	0.151E−05	2
8	37	38	684	761925.725	0.119E−06	4
9	507	508	1016	428.056916	0.347E−13	4
10	109	127	990	−80.4518214	0.639E−06	4
11	6	8	42	72291.4951	0.178E−08	4
12	519	520	2080	4994.21410	0.236E−06	4
13	3	4	16	0.660542076E−23	0.363E−11	3
14	2	3	18	0.129013604E−08	0.792E−06	4
15	9	10	40	1.92401599	0.414E−06	4
16	15	18	96	−427.391653	0.342E−06	4
17	8	9	54	−0.379921091E−01	0.314E−10	4
18	7	8	48	−0.245741193E−01	0.218E−09	4
19	13	16	84	1654.94525	0.174E−08	4
20	14	15	90	−1.00013520	0.139E−08	4
21	9	10	60	2.41354873	0.388E−08	4
22	30	34	186	1.00000000	0.164E−08	4
Σ	2919	2974	12442		TIME = 6.56	

Table 17: Results obtained by program TNEDS

Problem	NIT	NFV	NFG	F	G	ITERM
1	1447	1450	5792	0.173249493E−16	0.138E−06	3
2	79	89	400	0.169144088E−20	0.382E−09	3
3	18	19	114	0.180692317E−09	0.316E−06	4
4	24	25	100	269.499543	0.136E−08	4
5	11	12	72	0.990922474E−10	0.511E−06	4
6	17	21	252	0.166904871E−10	0.898E−06	4
7	11	12	72	336.937181	0.629E−06	4
8	6	11	126	761774.954	0.237E−05	2
9	7	8	16	316.436141	0.362E−08	4
10	70	74	639	−133.630000	0.221E−07	4
11	71	72	432	10.7765879	0.237E−10	4
12	133	134	536	982.273617	0.203E−07	4
13	7	8	32	0.402530175E−26	0.153E−13	3
14	2	3	18	0.129028794E−08	0.820E−06	4
15	10	11	44	1.92401599	0.217E−06	4
16	12	15	78	−427.404476	0.894E−09	4
17	8	9	54	−0.379921091E−01	0.391E−09	4
18	8	9	54	−0.245741193E−01	0.705E−10	4
19	7	8	48	59.5986241	0.106E−08	4
20	10	11	66	−1.00013520	0.277E−11	4
21	11	12	72	2.13866377	0.154E−06	4
22	46	51	282	1.00000000	0.376E−08	4
Σ	2015	2064	9299	NCG = 1182	TIME = 2.92	

Table 18: Results obtained by program TNECU

Problem	NIT	NFV	NFG	F	G	ITERM
1	1433	1438	5736	0.000000000	0.000E−00	3
2	218	241	1085	3918.57298	0.377E+01	4
3	17	19	108	0.191263604E−09	0.349E−06	4
4	10	12	44	269.522686	0.733E−08	4
5	13	15	84	0.309044401E−13	0.194E−06	4
6	13	14	196	0.328095928E−12	0.411E−06	4
7	19	27	120	337.413070	0.438E−06	4
8	37	38	684	761925.725	0.536E−06	2
9	643	644	1288	428.056916	0.432E−13	4
10	124	151	1125	−79.4726101	0.531E−07	4
11	6	8	42	72291.4951	0.188E−08	4
12	199	200	800	4994.21410	0.238E−06	4
13	4	5	20	0.650986116E−23	0.504E−11	3
14	2	3	18	0.129028794E−08	0.820E−06	4
15	10	11	44	1.92401599	0.217E−06	4
16	14	17	90	−427.391653	0.114E−12	4
17	8	9	54	−0.379921091E−01	0.391E−09	4
18	8	9	54	−0.245741193E−01	0.705E−10	4
19	13	16	84	1654.94525	0.155E−08	4
20	10	11	66	−1.00013520	0.277E−11	4
21	9	10	60	2.41354873	0.517E−06	4
22	46	51	282	1.00000000	0.376E−08	4
Σ	2900	2989	12304	NCG = 1877	TIME = 6.17	

Table 19: Results obtained by program TNECS

Problem	NIT	NFV	NFG	F	G	ITERM
1	2654	3627	3627	0.794789730E−16	0.213E−06	3
2	105	179	179	83.3161404	0.498E−06	4
3	40	45	45	0.267007684E−12	0.823E−06	4
4	37	45	45	269.499543	0.605E−06	4
5	16	17	17	0.106026711E−11	0.728E−06	4
6	38	40	40	0.546961387E−11	0.882E−06	4
7	22	26	26	335.252624	0.105E−06	4
8	26	40	40	761774.954	0.295E−04	2
9	193	202	202	316.436141	0.155E−05	2
10	227	258	258	−125.810000	0.351E−04	2
11	100	127	127	10.7765879	0.566E−06	4
12	28	29	29	982.273617	0.102E−06	4
13	1	2	2	0.00000000	0.000E+00	3
14	25	28	28	0.104289352E−08	0.927E−06	4
15	8	15	15	1.92401599	0.482E−07	4
16	25	35	35	−427.404476	0.130E−06	4
17	15	17	17	−0.379921091E−01	0.141E−06	4
18	5	11	11	−0.245741193E−01	0.311E−07	4
19	19	23	23	59.5986241	0.466E−06	4
20	37	97	97	−1.00013520	0.212E−08	4
21	37	40	40	2.13866377	0.767E−06	4
22	55	211	211	1.00000000	0.610E−07	4
Σ	3713	5114	5114		TIME = 4.27	

Table 20: Results obtained by program TSEDU

Problem	NIT	NFV	NFG	F	G	ITERM
1	2591	3322	3322	0.00000000	0.000E+00	3
2	344	347	347	35.1211309	0.107E−06	4
3	39	43	43	0.441691821E−12	0.425E−06	4
4	21	22	22	269.522686	0.105E−06	4
5	16	17	17	0.783032535E−11	0.279E−06	4
6	32	33	33	0.959526458E−11	0.801E−06	4
7	19	21	21	337.722479	0.247E−06	4
8	52	56	56	761925.725	0.780E−04	2
9	1001	1003	1003	428.056916	0.192E−06	4
10	191	222	222	−86.7038382	0.225E−05	2
11	13	18	18	72291.4951	0.285E−08	4
12	228	235	235	4994.21410	0.304E−06	4
13	1	2	2	0.00000000	0.000E+00	3
14	25	28	28	0.104289352E−08	0.927E−06	4
15	8	15	15	1.92401599	0.534E−07	4
16	21	22	22	−427.391653	0.759E−06	4
17	15	17	17	−0.379921091E−01	0.299E−06	4
18	5	10	10	−0.245741193E−01	0.193E−07	4
19	20	25	25	1654.94525	0.351E−06	4
20	78	130	130	−1.00013520	0.196E−06	4
21	27	31	31	2.41354873	0.202E−06	4
22	52	190	190	1.00000000	0.418E−06	4
Σ	4799	5809	5809		TIME = 7.68	

Table 21: Results obtained by program TSEDS

Problem	NIT	NFV	NFG	F	G	ITERM
1	2606	3566	3566	0.650526028E−17	0.969E−07	3
2	108	177	177	111.363179	0.853E−05	2
3	40	45	45	0.267007684E−12	0.823E−06	4
4	37	45	45	269.499543	0.605E−06	4
5	16	17	17	0.106026711E−11	0.728E−06	4
6	38	40	40	0.546961387E−11	0.882E−06	4
7	26	31	31	335.252624	0.315E−06	4
8	25	39	39	761774.954	0.149E−03	2
9	191	210	210	316.436141	0.115E−05	2
10	233	264	264	−121.691827	0.190E−04	2
11	113	144	144	10.7765879	0.649E−07	4
12	28	29	29	982.273617	0.103E−06	4
13	1	2	2	0.00000000	0.000E+00	3
14	25	28	28	0.104289348E−08	0.927E−06	4
15	27	41	41	1.92401599	0.668E−07	4
16	25	35	35	−427.404476	0.263E−06	4
17	15	17	17	−0.379921091E−01	0.141E−06	4
18	8	12	12	−0.245741193E−01	0.358E−11	4
19	19	23	23	59.5986241	0.466E−06	4
20	42	74	74	−1.00013520	0.965E−09	4
21	37	40	40	2.13866377	0.767E−06	4
22	48	192	192	1.00000000	0.107E−06	4
Σ	3708	5071	5071	NCG = 48642	TIME = 7.63	

Table 22: Results obtained by program TSECU

Problem	NIT	NFV	NFG	F	G	ITERM
1	2598	3347	3347	0.00000000	0.000E+00	3
2	352	361	361	35.1211309	0.853E−05	2
3	39	43	43	0.441691822E−12	0.425E−06	4
4	21	22	22	269.522686	0.105E−06	4
5	16	17	17	0.783032535E−11	0.279E−06	4
6	32	33	33	0.959526458E−11	0.801E−06	4
7	19	21	21	337.722479	0.162E−05	2
8	46	49	49	761925.725	0.792E−04	2
9	1001	1003	1003	428.056916	0.348E−08	4
10	203	233	233	−86.7188428	0.288E−04	2
11	21	38	38	72291.4951	0.135E−10	4
12	223	230	230	4994.21410	0.303E−06	4
13	1	2	2	0.00000000	0.000E+00	3
14	25	28	28	0.104289348E−08	0.927E−06	4
15	17	27	27	1.92401599	0.553E−07	4
16	21	22	22	−427.391653	0.759E−06	4
17	15	17	17	−0.379921091E−01	0.299E−06	4
18	8	12	12	−0.245741193E−01	0.358E−11	4
19	20	25	25	1654.94525	0.351E−06	4
20	33	46	46	−1.00013520	0.959E−10	4
21	27	31	31	2.41354873	0.202E−06	4
22	51	185	185	1.00000000	0.834E−06	4
Σ	4789	5792	5792	NCG = 15187	TIME = 6.32	

Table 23: Results obtained by program TSECS

Problem	NIT	NFV	NFG	F	G	ITERM
1	3124	3134	3134	0.287703261E−08	0.582E−08	4
2	286	287	287	0.379499216E−08	0.203E−06	2
3	71	71	71	0.233196848E−09	0.100E−07	4
4	40	40	40	126.863549	0.699E−08	4
5	282	282	282	0.732927514E−07	0.400E−08	4
6	344	344	344	0.836329152E−08	0.326E−08	4
7	286	287	287	2391.16999	0.673E−04	2
8	610	611	611	0.317244739E−05	0.548E−08	4
9	2514	2516	2516	552.380551	0.448E−08	4
10	907	907	907	131.888476	0.579E−08	4
11	269	271	271	0.173668302E−09	0.266E−08	4
12	1805	1810	1810	621.128947	0.906E−02	2
13	680	681	681	2940.50943	0.140E−03	2
14	370	370	370	112.314954	0.622E−08	4
15	364	364	364	36.0935676	0.986E−08	4
16	1004	1004	1004	13.2000000	0.904E−08	4
17	380	380	380	0.268534232E−01	0.871E−09	4
18	15319	15321	15321	0.589970806E−08	0.925E−08	4
19	3972	4056	4056	0.565862690E−08	0.887E−08	4
20	774	988	988	0.406495193E−08	0.468E−08	4
21	247	248	248	264.000000	0.364E−03	2
22	1191	1192	1192	593.360762	0.145E−03	2
Σ	34839	35164	35164		TIME = 13.49	

Table 24: Results obtained by program TSENU

Problem	NIT	NFV	NFG	F	G	ITERM
1	1377	1379	1379	0.697391982E−22	0.130E−09	3
2	41	46	46	0.216572157E−16	0.154E−06	3
3	11	12	14	0.136731713E−09	0.233E−06	4
4	13	16	21	134.749772	0.279E−06	4
5	4	5	7	0.111058357E−10	0.887E−06	4
6	6	7	13	0.742148235E−26	0.303E−12	3
7	10	12	23	60734.8551	0.648E−07	4
8	21	26	24	0.253357740E−08	0.800E−06	4
9	15	16	36	2216.45871	0.104E−10	4
10	12	18	21	191.511336	0.524E−07	4
11	2587	2593	2649	0.647358980E−27	0.359E−12	3
12	16	20	23	19264.6341	0.513E−10	4
13	17	21	28	131234.018	0.784E−08	4
14	5	8	18	108.517888	0.227E−08	4
15	6	7	15	18.1763146	0.290E−06	4
16	15	21	40	2.51109677	0.724E−06	4
17	15	20	19	0.257973699E−16	0.275E−08	3
18	42	44	45	0.151517993E−24	0.122E−10	3
19	15	16	23	0.354943701E−14	0.255E−06	4
20	26	27	29	0.378161520E−10	0.407E−07	4
21	10	11	17	647.828517	0.773E−11	4
22	26	32	45	4486.97024	0.602E−07	4
Σ	4290	4357	4535		TIME = 4.56	

Table 25: Results obtained by program TGADU

Problem	NIT	NFV	NFG	F	G	ITERM
1	1011	1013	1013	0.00000000	0.000E+00	3
2	260	273	508	1959.28649	0.439E−12	4
3	10	12	13	0.784354965E−09	0.868E−06	4
4	14	18	19	134.761343	0.827E−08	4
5	4	5	7	0.438081882E−11	0.697E−06	4
6	6	7	13	0.791460684E−17	0.934E−08	3
7	22	23	61	145814.000	0.000E+00	4
8	25	32	28	0.978141069E−06	0.782E−06	4
9	44	45	153	2220.17880	0.181E−09	4
10	12	19	21	191.511336	0.301E−07	4
11	3977	2992	2990	0.00000000	0.000E+00	3
12	29	30	50	67887.2385	0.438E−12	4
13	19	20	36	147906.000	0.000E+00	4
14	1	2	6	126.690556	0.000E+00	4
15	24	27	81	18.1763146	0.203E−10	4
16	46	50	135	3.59074140	0.470E−10	4
17	11	12	15	0.969524252E−21	0.171E−10	3
18	0	1	3	0.00000000	0.000E+00	3
19	26	30	34	0.202602070E−14	0.193E−06	4
20	929	930	2780	498.800124	0.359E−05	2
21	20	21	33	649.598077	0.280E−08	4
22	24	31	55	4488.96148	0.242E−07	4
Σ	6514	5593	8054		TIME = 7.77	

Table 26: Results obtained by program TGADS

Problem	NIT	NFV	NFG	F	G	ITERM
1	1108	1110	1110	0.00000000	0.000E+00	3
2	624	640	649	66.4089431	0.283E−07	4
3	11	12	14	0.202412411E−09	0.210E−06	4
4	11	13	17	134.749772	0.592E−07	4
5	4	5	7	0.116836300E−10	0.908E−06	4
6	6	7	13	0.787821743E−26	0.311E−12	3
7	17	40	29	60734.8551	0.428E−05	6
8	22	25	25	0.127726626E−07	0.160E−06	4
9	13	15	38	2216.45871	0.846E−06	4
10	129	147	176	191.511336	0.104E−07	4
11	3010	3016	3012	0.402368464E−24	0.902E−11	3
12	205	226	236	22287.9069	0.449E−08	4
13	123	132	152	131234.018	0.743E−09	4
14	7	8	32	108.517888	0.148E−07	4
15	13	20	42	18.1763146	0.445E−05	2
16	14	15	35	2.51109677	0.103E−09	4
17	29	34	33	0.139780007E−09	0.238E−06	4
18	49	53	52	0.119511868E−21	0.344E−09	3
19	15	16	23	0.339085307E−13	0.788E−06	4
20	17	18	32	0.336618309E−11	0.137E−07	4
21	15	18	23	647.696136	0.262E−06	4
22	47	59	98	4486.97024	0.663E−08	4
Σ	5489	5629	5848	NCG = 8282	TIME = 5.01	

Table 27: Results obtained by program TGACU

Problem	NIT	NFV	NFG	F	G	ITERM
1	1028	1031	1030	0.00000000	0.000E+00	3
2	263	262	511	1959.28649	0.819E−10	4
3	10	12	13	0.814133878E−09	0.897E−06	4
4	11	14	17	134.761343	0.206E−10	4
5	4	5	7	0.438081882E−11	0.697E−06	4
6	6	7	13	0.791460667E−17	0.934E−08	3
7	15	16	42	145814.000	0.000E+00	4
8	17	18	20	0.201167216E−07	0.162E−06	4
9	54	55	203	2220.17880	0.614E−10	4
10	95	105	126	191.511336	0.527E−06	4
11	4577	3591	3587	0.00000000	0.000E+00	3
12	49	50	84	67887.2385	0.351E−07	4
13	18	19	41	147906.000	0.000E+00	4
14	1	2	6	126.690556	0.000E+00	4
15	33	72	85	18.1763146	0.182E−04	6
16	8	12	29	3.59074140	0.542E−06	4
17	25	29	29	0.716457302E−10	0.922E−07	4
18	0	1	3	0.00000000	0.000E+00	3
19	28	32	36	0.209831733E−13	0.620E−06	4
20	937	938	2806	498.800124	0.572E−12	4
21	21	22	34	649.598077	0.324E−08	4
22	44	51	89	4488.96148	0.645E−10	4
Σ	7244	6344	8811	NCG = 11206	TIME = 6.56	

Table 28: Results obtained by program TGACS

Problem	NIT	NFV	NFG	C	G	ITERM
1	53	66	54	0.260681654E−14	0.120E−07	4
2	107	160	108	0.502441303E−12	0.262E−06	4
3	33	41	34	0.535439934E−08	0.814E−06	4
4	51	93	52	0.540217976	0.383E−06	4
5	23	24	24	0.132910215E−08	0.482E−06	4
6	46	52	47	0.216701250E−08	0.436E−06	4
7	48	113	49	0.260162540	0.430E−06	4
8	21	58	22	282.380956	0.806E−06	4
9	59	146	60	0.185849706	0.287E−06	4
10	159	215	160	−0.251638288	0.250E−06	4
11	70	96	71	0.538829394E−01	0.139E−07	4
12	136	245	137	0.941962422	0.207E−06	4
13	2	4	3	0.456380111E−19	0.304E−11	3
14	5	6	6	0.162409086E−08	0.671E−06	4
15	116	120	117	0.199003538E−01	0.436E−06	4
16	110	214	111	−0.388943896E−02	0.518E−05	2
17	33	49	34	−0.110417781E−06	0.764E−06	4
18	62	77	63	0.744234285E−09	0.611E−07	4
19	9	23	10	42.6746811	0.373E−09	4
20	25	32	26	−0.497512435E−02	0.422E−06	4
21	16	23	17	0.298487756E−01	0.595E−06	4
22	32	82	33	0.577532726E−02	0.972E−07	4
Σ	1216	1939	1238		TIME = 0.75	

Table 29: Results obtained by program TMAXU

Problem	NIT	NFV	NFG	C	G	ITERM
1	337	355	338	0.193178806E−13	0.254E−05	3
2	127	151	128	0.120336380E−12	0.424E−05	3
3	25	28	26	0.383710546E−09	0.331E−06	4
4	67	77	68	126.863549	0.358E−02	2
5	6	7	7	0.494049246E−14	0.666E−07	3
6	13	17	14	0.663150090E−13	0.141E−06	3
7	73	108	74	2391.16999	0.209E+00	2
8	241	243	242	0.383133726E−07	0.708E−06	4
9	209	251	209	552.682636	0.267E+01	−6
10	84	106	85	131.888475	0.242E−05	2
11	732	751	733	0.799693645E−12	0.581E−05	3
12	203	237	204	612.723020	0.601E−04	2
13	90	111	91	2940.50941	0.245E−03	2
14	84	107	85	112.314955	0.480E−05	2
15	39	64	40	36.0935678	0.163E−01	2
16	67	108	67	13.2000005	0.139E−03	6
17	337	344	338	0.100472795E−13	0.167E−06	3
18	3637	3794	3638	0.199840144E−14	0.419E−09	3
19	23	24	24	0.938360500E−12	0.217E−04	3
20	22	44	22	0.121058719E−11	0.398E−05	6
21	65	90	66	262.921649	0.108E−05	2
22	608	627	609	593.367735	0.525E−02	2
Σ	7089	7644	7108		TIME = 2.80	

Table 30: Results obtained by program TSUMU

Problem	NIT	NFV	NFG	F	G	ITERM
1	10	41	0	0.224531E−22	0.168207E−07	3
2	9	46	0	0.106897E−22	0.163517E−06	3
3	3	19	0	0.333989E−19	0.223053E−06	3
4	7	23	0	0.348196E−17	0.177085E−02	3
5	12	63	0	0.117206E−16	0.694210E−06	3
6	17	52	0	0.110919E−16	0.167579E−11	3
7	13	41	0	0.339913E−19	0.457009E−03	3
8	13	73	0	0.125748E−25	0.193922E−04	3
9	13	99	0	0.432936E−21	0.201706E−03	3
10	5	41	0	0.803846E−25	0.415983E−03	3
11	12	37	0	0.189327E−25	0.423583E−05	3
12	18	55	0	0.129272E−16	0.713317E−13	3
13	18	39	0	0.105290E−16	0.341327E−13	3
14	4	13	0	0.774783E−20	0.441968E−05	3
15	5	36	0	0.182567E−17	0.471251E−03	3
16	53	319	0	0.462169E−17	0.153957	3
17	14	48	0	0.449140E−22	0.105525E−03	3
18	26	79	0	0.977445E−16	0.245792E−01	3
19	2	7	0	0.309324E−21	0.370062E−09	3
20	13	43	0	0.428279E−20	0.203421E−07	3
21	12	37	0	0.200623E−20	0.255404E−10	3
22	7	50	0	0.195350E−19	0.106707E−05	3
23	29	262	0	0.390327E−17	0.200697E−10	3
24	6	31	0	0.822526E−23	0.812457E−09	3
25	9	46	0	0.147127E−23	0.395357E−09	3
26	12	61	0	0.608837E−17	0.420862E−07	3
27	10	51	0	0.275078E−20	0.121824E−06	3
28	10	60	0	0.229532E−16	0.213811E−05	3
29	4	53	0	0.124549E−19	0.130673E−05	3
30	12	162	0	0.222959E−21	0.107876E−07	3
Σ	378	1987	0	NCG = 1543	TIME = 3.81	

Table 31: Results obtained by program TEQNU

Problem	NIT	NFV	NFG	F	G	ITERM
1	30	64	0	0.326079E−18	0.154142E−03	3
2	17	57	0	0.720058E−19	0.261551E−07	3
3	5	11	0	0.861220E−16	0.366389E−03	3
4	11	19	0	0.115060E−18	0.358897E−01	3
5	20	56	0	0.335602E−16	0.121910E−06	3
6	22	31	0	0.167377E−16	0.898624E−08	3
7	25	42	0	0.137004E−20	0.185851E−05	3
8	21	60	0	0.496243E−28	0.183782E−07	3
9	32	71	0	0.220876E−21	0.800603E−05	3
10	9	24	0	0.202316E−20	0.162996E−03	3
11	16	23	0	0.116022E−21	0.130018E−02	3
12	23	40	0	0.861690E−16	0.190460E−08	3
13	24	32	0	0.234892E−16	0.204525E−08	3
14	8	13	0	0.596974E−21	0.811563E−05	3
15	12	28	0	0.124901E−17	0.305897	3
16	22	78	0	0.984840E−20	0.125407E−03	3
17	17	43	0	0.130235E−20	0.154659E−04	3
18	46	61	0	0.224793E−17	0.116353E−01	3
19	2	5	0	0.704403E−18	0.221630E−06	3
20	18	30	0	0.158787E−16	0.312477E−03	3
21	25	34	0	0.233925E−16	0.135133E−05	3
22	14	45	0	0.189862E−17	0.128826E−01	3
23	23	106	0	0.194742E−18	0.550497E−08	3
24	20	53	0	0.737500E−17	0.611156E−08	3
25	29	50	0	0.208794E−17	0.413643E−08	3
26	36	67	0	0.132055E−17	0.481013E−08	3
27	40	75	0	0.659356E−17	0.862034E−08	3
28	27	83	0	0.461856E−18	0.268680E−08	3
29	12	95	0	0.206962E−16	0.754042E−08	3
30	18	145	0	0.740533E−16	0.167985E−07	3
Σ	624	1541	0	NCG = 1332	TIME = 3.22	

Table 32: Results obtained by program TEQLU

References

[1] Al-Baali M., Fletcher R.: Variational methods for nonlinear least squares. Journal of Optimization Theory and Applications 36 (1985) 405-421.

[2] Brown, P.N., Saad, Y.: Convergencet theory of nonlinear Newton-Krylov algorithms. SIAM Journal on Optimization 4 (1994) 297-330.

[3] Byrd R.H., Nocedal J., Schnabel R.B.: Representation of quasi-Newton matrices and their use in limited memory methods. Math. Programming 63 (1994) 129-156.

[4] Coleman T.F., and Moré J.J.: Estimation of sparse Jacobian and graph coloring problem. SIAM Journal on Numerical Analysis 20, (1983) 187-209.

[5] Coleman, T.F., Moré J.J.: Estimation of sparse Hessian matrices and graph coloring problems. Mathematical Programming 28 (1984) 243-270.

[6] Curtis, A.R., Powell, M.J.D., and Reid, J.K.: On the estimation of sparse Jacobian matrices. IMA Journal of Aplied Mathematics 13 (1974) 117-119.

[7] Dembo, R.S, Eisenstat, S.C., and Steihaug T.: Inexact Newton methods. SIAM J. on Numerical Analysis 19 (1982) 400-408.

[8] Dembo, R.S, Steihaug T.: Truncated Newton algorithms for large-scale optimization. Math. Programming 26 (1983) 190-212.

[9] Dennis J.E., Mei H.H.W: An unconstrained optimization algorithm which uses function and gradient values. Report No. TR 75-246, 1975.

[10] Gill P.E., Murray W.: Newton type methods for unconstrained and linearly constrained optimization. Math. Programming 7 (1974) 311-350.

[11] Gould N.I.M, Lucidi S., Roma M., Toint P.L.: Solving the trust-region subproblem using the Lanczos method. Report No. RAL-TR-97-028, 1997.

[12] Griewank A., Toint P.L.: Partitioned variable metric updates for large-scale structured optimization problems. Numer. Math. 39 (1982) 119-137.

[13] Liu D.C., Nocedal J.: On the limited memory BFGS method for large scale optimization. Math. Programming 45 (1989) 503-528.

[14] Lukšan L.: Combined trust region methods for nonlinear least squares. Kybernetika 32 (1996) 121-138.

[15] Lukšan L.: Hybrid methods for large sparse nonlinear least squares. J. Optimizaton Theory and Applications 89 (1996) 575-595.

[16] Lukšan L., Matonoha C., Vlček J.: A shifted Steihaug-Toint method for computing a trust-region step Report V-914, Prague, ICS AS CR, 2004.

[17] Lukšan L., Matonoha C., Vlček J.: Primal interior-point method for large sparse minimax optimization. Technical Report V-941, Prague, ICS AS CR, 2005.

[18] Lukšan L., Matonoha C., Vlček J.: Trust-region interior point method for large sparse l_1 optimization. Technical Report V-942, Prague, ICS AS CR, 2005.

[19] Lukšan L., Spedicato E.: Variable metric methods for unconstrained optimization and nonlinear least squares. Journal of Computational and Applied Mathematics 124 (2000) 61-93.

[20] Lukšan L., Tůma M., Hartman J., Vlček J., Ramešová N., Šiška M., Matonoha C.: Interactive System for Universal Functional Optimization (UFO). Version 2006. Technical Report V-977. Prague, ICS AS CR 2006.

[21] Lukšan L., Vlček J. Sparse and partially separable test problems for unconstrained and equality constrained optimization. Report V-767, Prague, ICS AS CR, 1998.

[22] Lukšan L., Vlček J.: Computational Experience with Globally Convergent Descent Methods for Large Sparse Systems of Nonlinear Equations. Optimization Methods and Software 8 (1998) 201-223.

[23] Lukšan L., Vlček J.: Variable metric method for minimization of partially separable nonsmooth functions. Pacific Journal on Optimization 2 (2006).

[24] Martinez, J.M., and Zambaldi, M.C.: An Inverse Column-Updating Method for Solving Large-Scale Nonlinear Systems of Equations. Optimization Methods and Software 1 (1992) 129-140.

[25] Moré J.J., Sorensen D.C.: Computing a trust region step. SIAM Journal on Scientific and Statistical Computations 4 (1983) 553-572.

[26] Nocedal J.: Updating quasi-Newton matrices with limited storage. Math. Comp. 35 (1980) 773-782.

[27] Nowak U., Weimann L.: A family of Newton codes for systems of highly nonlinear equations. Tech. Report TR-91-10, Konrad-Zuse-Zentrum für Informationstechnik, Berlin 1991.

[28] Pernice M., Walker H.F.: NITSOL: A Newton iterative solver for nonlinear systems. SIAM J. on Scientific Computing 19 (1998) 302-318.

[29] Powell M.J.D: A new algorithm for unconstrained optimization. In: Nonlinear Programming (J.B.Rosen O.L.Mangasarian, K.Ritter, eds.) Academic Press, London 1970.

[30] Schlick T., Fogelson A.: TNPACK – A Truncated Newton Minimization Package for Large-Scale Problems (Parts I and II). ACM Transactions on Mathematical Software 18 (1992) 46-111.

[31] Steihaug T.: The conjugate gradient method and trust regions in large-scale optimization. SIAM Journal on Numerical Analysis 20 (1983) 626-637.

[32] Toint P.L.: Towards an efficient sparsity exploiting Newton method for minimization. In: Sparse Matrices and Their Uses (I.S.Duff, ed.), Academic Press, London 1981, 57-88.

[33] Toint P.L.: Subroutine VE08. Harwell Subroutine Library. Specifications (Release 12), Vol, 2, AEA Technology, December 1995, 1162-1174.

[34] Toint P.L.: Subroutine VE10. Harwell Subroutine Library. Specifications (Release 12), Vol, 2, AEA Technology, December 1995, 1187-1197.

[35] Tong C.H.: A comparative study of preconditioned Lanczos methods for nonsymmetric linear systems. Sandia National Laboratories, Sandia Report No. SAND91-8240B, Sandia National Laboratories, Livermore, 1992.

[36] Tůma M.: A note on direct methods for approximations of sparse Hessian matrices. Aplikace Matematiky 33 (1988) 171-176.

[37] Vlček J., Lukšan L.: Globally convergent variable metric method for nonconvex nondifferentiable unconstrained minimization. Journal of Optimization Theory and Applications 111 (2001) 407-430.

[38] Vlček J., Lukšan L.: Shifted limited-memory variable metric methods for large-scale unconstrained minimization. J. of Computational and Applied Mathematics, 186 (2006) 365-390.

[39] Watson L.T., Melville R.C., Morgan A.P., Walker H.F: Algorithm 777. HOMPACK90: A Suite of Fortran 90 Codes for Globally Convergent Homotopy Algorithms. ACM Transactions on Mathematical Software 23 (1997) 514-549.

[40] Zhu C., Byrd R.H., Lu P., Nocedal J.: Algorithm 778. L-BFGS-B: Fortran Subroutines for Large-Scale Bound Constrained Optimization. ACM Transactions on Mathematical Software 23 (1997) 550-560.
